# Supplementary material for: Local H2 release remodels senescence microenvironment for improved repair of injured bone
Source: Nat Commun. 2023 Nov 27;14:7783. doi: 10.1038/s41467-023-43618-z (PMC10682449; doi:10.1038/s41467-023-43618-z)
Supplement: Supplementary file 1 — Supplementary Information [file 41467_2023_43618_MOESM1_ESM.pdf]

Supplementary Information

Local H_2 release remodels senescence microenvironment for improved repair of injured bone

Shengqiang Chen,[1,2#] Yuanman Yu,[3#] Songqing Xie,[1#] Danna Liang,[4] Wei Shi,[1] Sizhen Chen,[1] Guanglin Li,[1] Wei Tang,[1]* Changsheng Liu,[3]* Qianjun He[2,5,6]*

[1] *Key Laboratory of Human-Machine-Intelligence Synergic System, Research Center for Neural Engineering, Shenzhen Institute of Advanced Technology, Chinese Academy of Sciences, Shenzhen 518055, Guangdong, China*

[2] *Shanghai Key Laboratory of Hydrogen Science & Center of Hydrogen Science, School of Materials Science and Engineering, Shanghai Jiao Tong University, Shanghai 200240, China*

[3] *The State Key Laboratory of Bioreactor Engineering, East China University of Science and Technology, Shanghai 200237, China*

[4] *Guangdong Key Laboratory for Biomedical Measurements and Ultrasound Imaging, School of Biomedical Engineering, Health Science Center, Shenzhen University, Shenzhen 518060, China*

[5] *Medical Center on Aging, Ruijin Hospital, Shanghai Jiao Tong University School of Medicine, Shanghai 200025, China*

[6] *Shenzhen Research Institute, Shanghai Jiao Tong University, Shenzhen 518057, China*

# These authors contributed equally to this work.

* Correspondence should be addressed to W.T. (email: wei.tang1@siat.ac.cn), C.L. (email: liucs@ecust.edu.cn), and Q.H. (email: nanoflower@126.com).

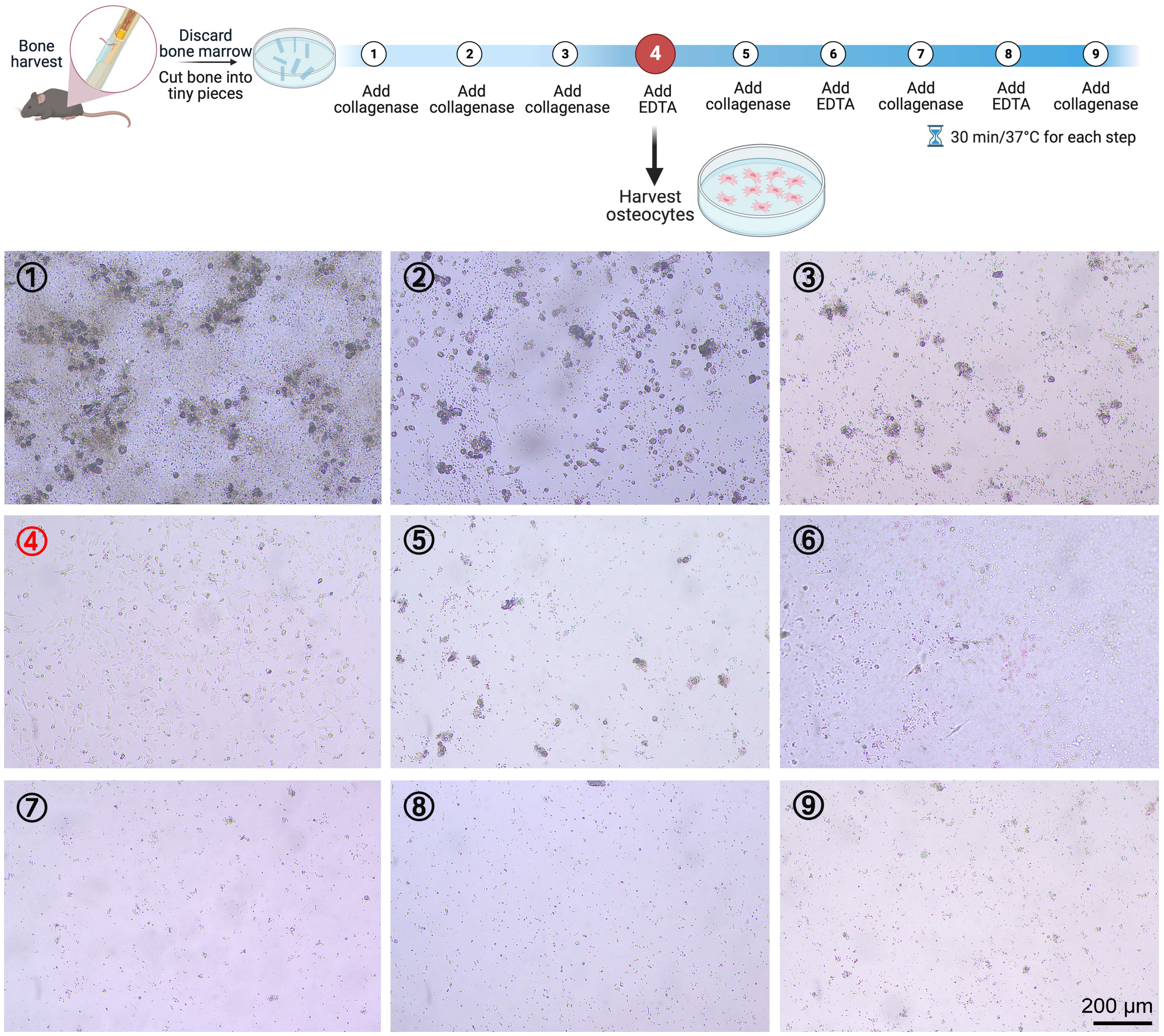

Supplementary Figure 1. Schematic illustration of the protocol of osteocytes isolation. The schematic is created with BioRender.com. The cells at step ④ represented a highly enriched population of osteocytes, and were therefore collected and used in this study. The experiments were repeated four times independently with similar results.

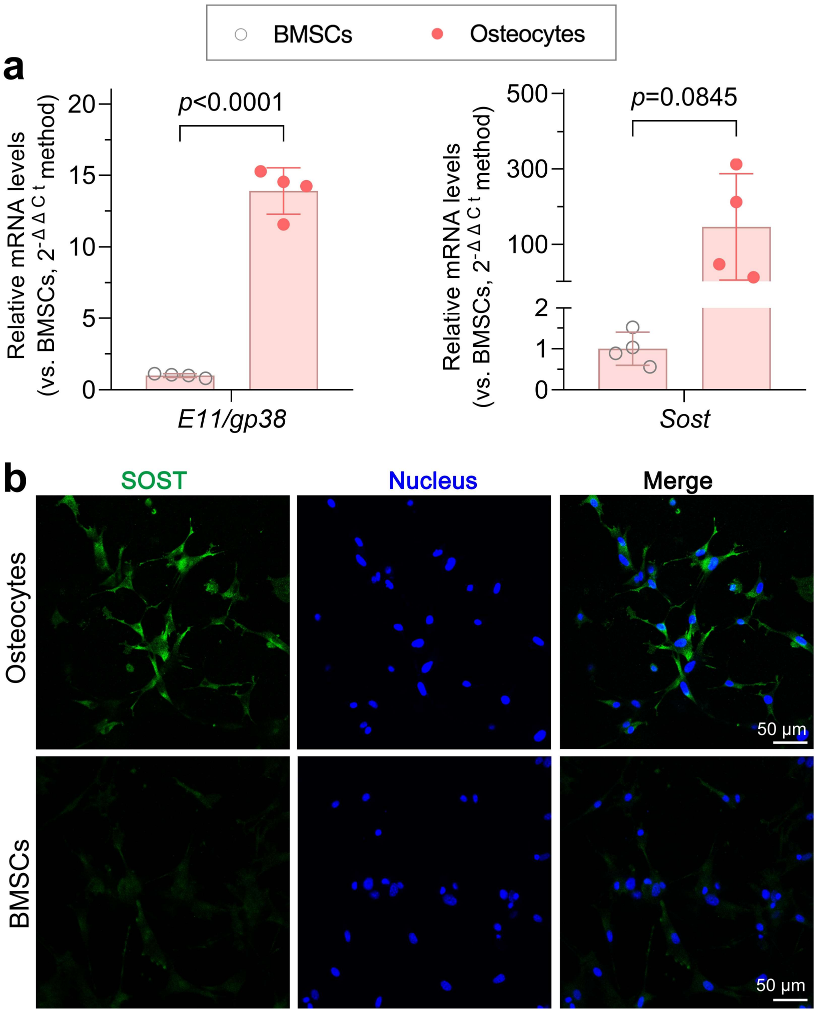

Supplementary Figure 2. (**a**) Quantitative reverse transcription-polymerase chain reaction (qRT-PCR) analysis of osteocyte markers (*E11/gp38* and *Sost*) ($n = 4$, biologically independent samples). (**b**) Immunofluorescence staining of sclerostin (SOST, a marker of mature osteocytes). The experiments were repeated four times independently with similar results. Compared with BMSCs, osteocytes displayed a characteristic osteocyte-like dendritic morphology and expressed a higher level of osteocyte marker. Data are means ± SD. $^{****}p < 0.0001$ [Two-tailed unpaired Student's *t* test].

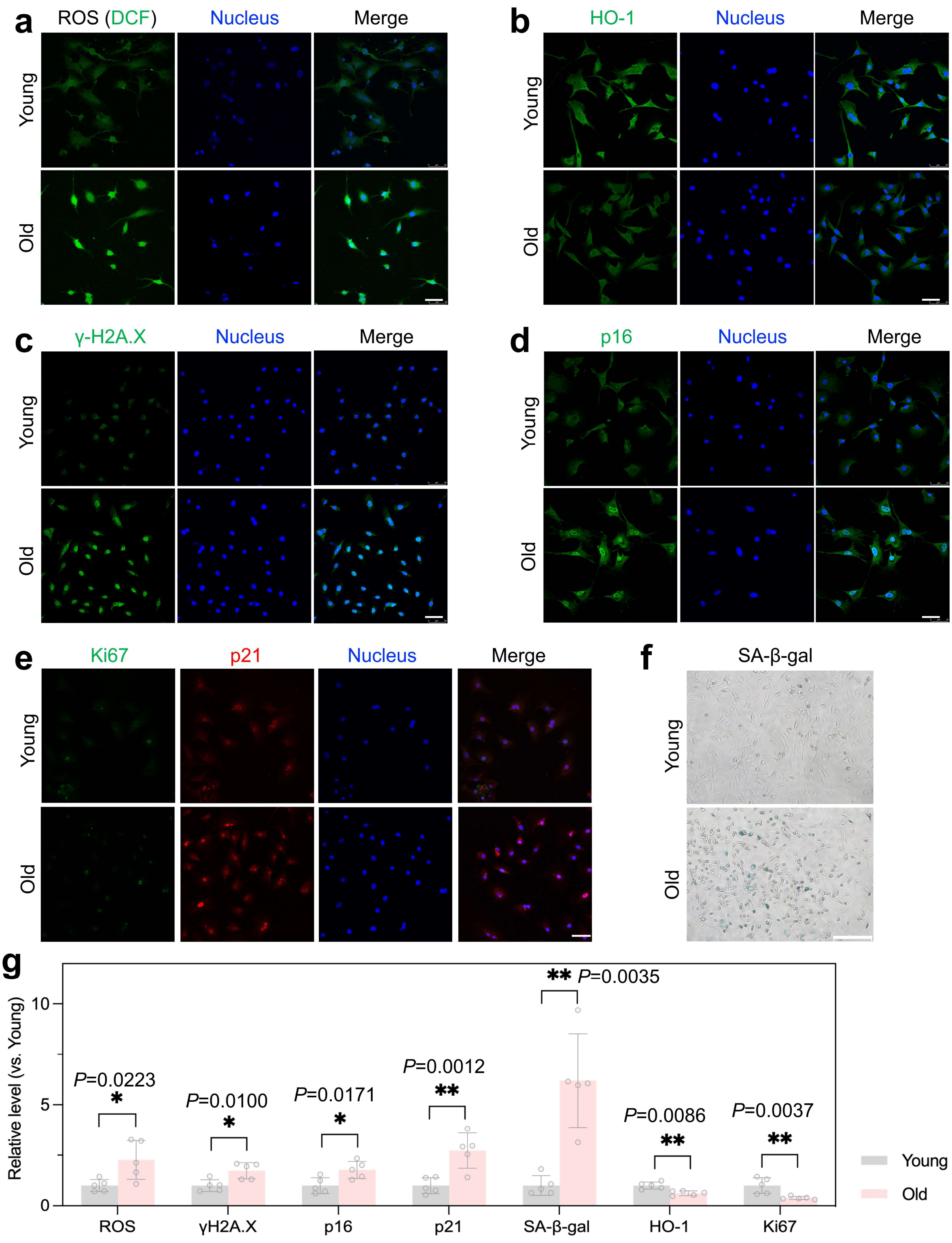

Supplementary Figure 3. The senescence characteristics of osteocytes harvested from young (from 6-week-old mice) and aging (from 24-month-old mice) bone after 7 days of culture under general conditions. Representative images of (**a**) ROS levels (DCF), (**b**) HO-1, (**c**) γ-H2A.X, (**d**) p16, (**e**) Ki67/p21, (**f**) SA-β-gal, and (**g**) relative levels of the senescence-related markers (n = 5, biologically independent samples). Scale bars a–e and f represent 50 μm and 200 μm, respectively. Data are means ± SD. *$p < 0.05$, **$p < 0.01$ [Two-tailed unpaired Student's t test].

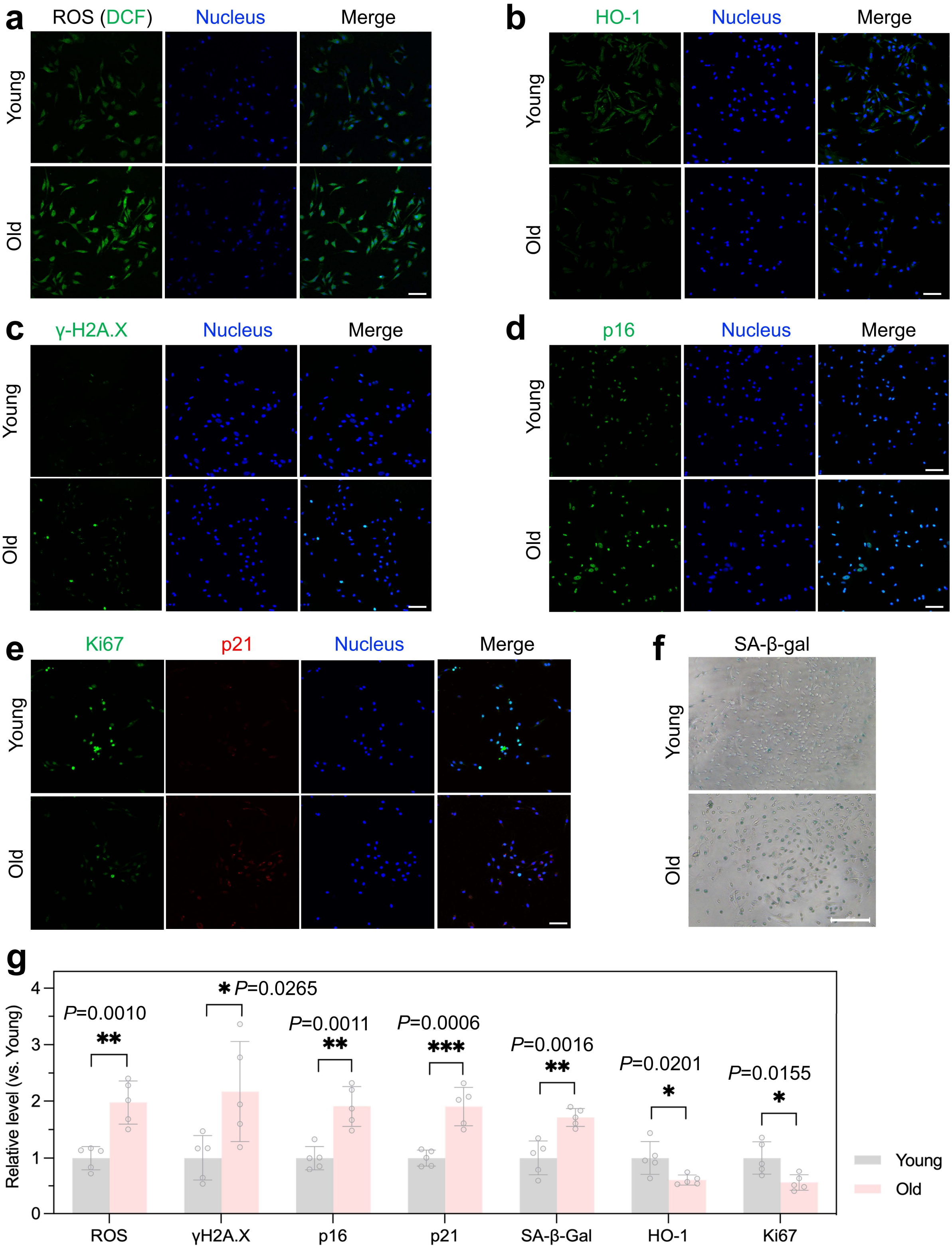

Supplementary Figure 4. The senescence characteristics of BMSCs harvested from young (from 6-week-old mice) and aging (from 24-month-old mice) bone after 7 days of culture under general conditions. Representative images of (**a**) ROS levels (DCF), (**b**) HO-1, (**c**) γ-H2A.X, (**d**) p16, (**e**) Ki67/p21, (**f**) SA-β-gal, and (**g**) relative levels of the senescence-related markers ($n = 5$, biologically independent samples). Scale bars a–e and f represent 50 μm and 200 μm, respectively. Data are means ± SD. $*p < 0.05$, $**p < 0.01$, $***p < 0.001$ [Two-tailed unpaired Student's t test].

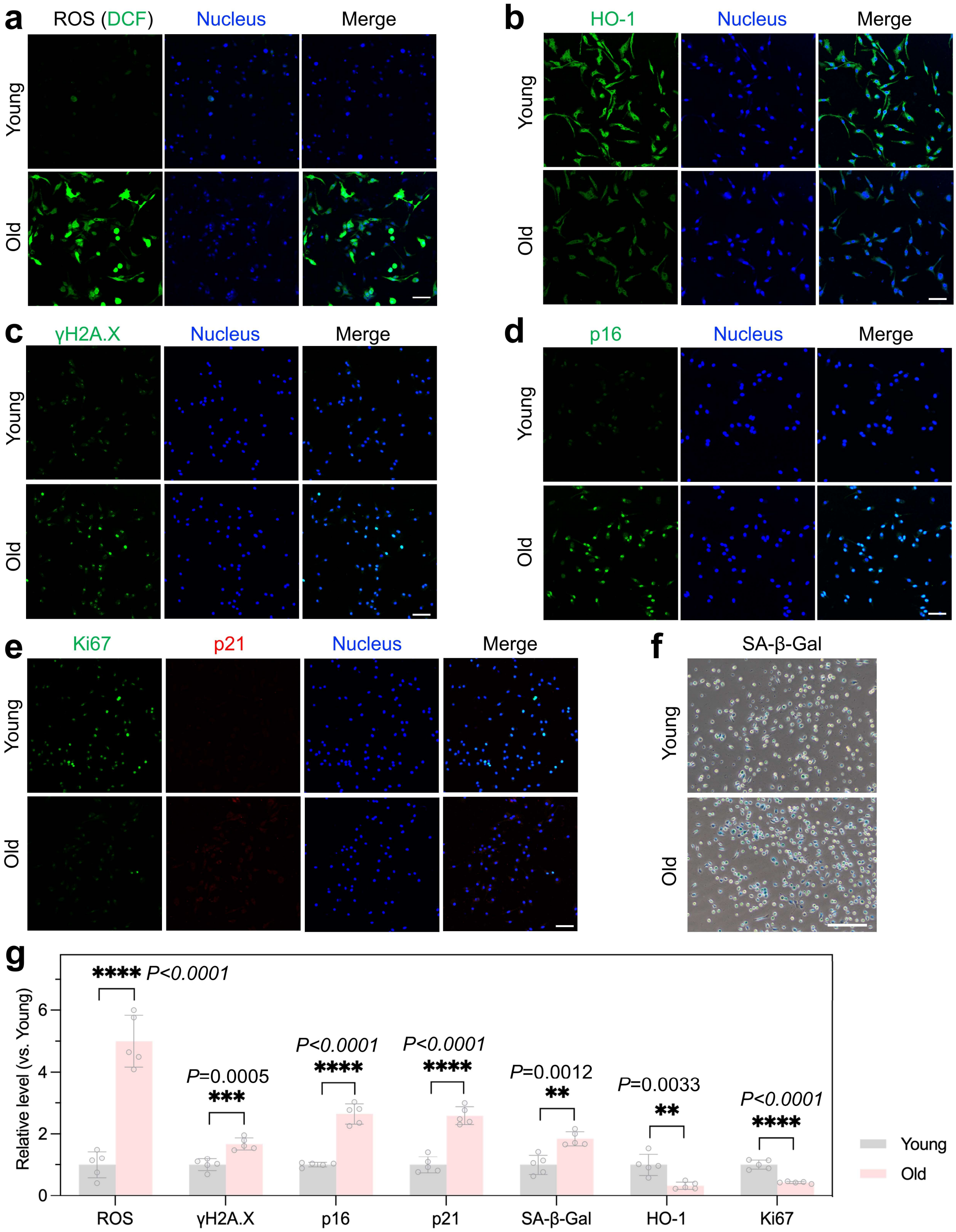

Supplementary Figure 5. The senescence characteristics of macrophages harvested from young (from 6-week-old mice) and aging (from 24-month-old mice) bone after 7 days of culture under general conditions. Representative images of (**a**) ROS levels (DCF), (**b**) HO-1, (**c**) γ-H2A.X, (**d**) p16, (**e**) Ki67/p21, (**f**) SA-β-gal, and (**g**) relative levels of the senescence-related markers (n = 5, biologically independent samples). Scale bars a–e and f represent 50 μm and 200 μm, respectively. Data are means ± SD. $^{**}p < 0.01$, $^{***}p < 0.001$, and $^{****}p < 0.0001$ [Two-tailed unpaired Student's t test].

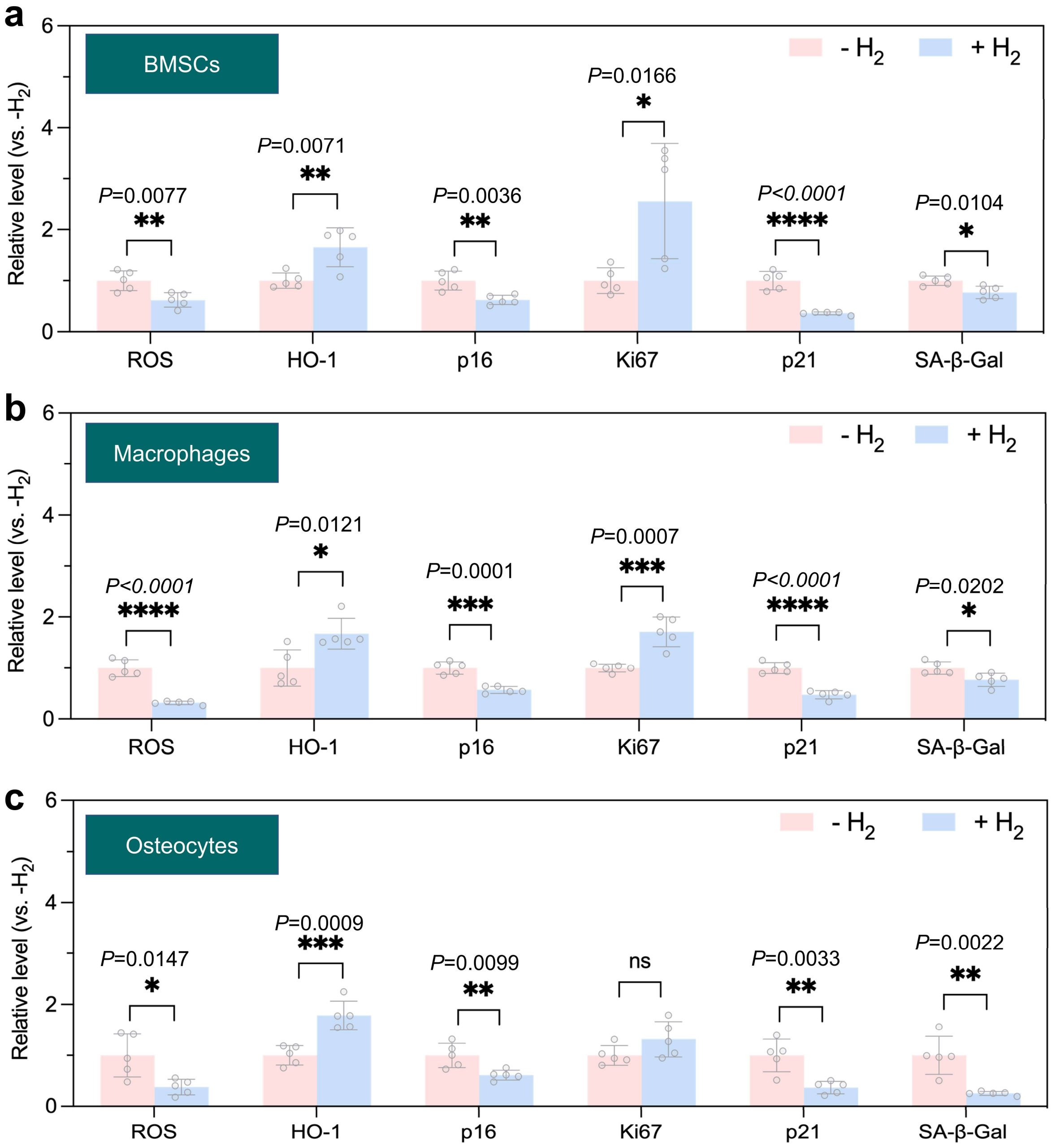

Supplementary Figure 6. Quantitative analysis of the senescence-related markers in H_2-treated BMSCs (**a**), macrophages (**b**) and osteocytes (**c**) harvested from aging (from 24-month-old mice) bone relative to the ones without H_2 treatment ($n = 5$, biologically independent samples). Data are means ± SD. *$p < 0.05$, **$p < 0.01$, ***$p < 0.001$, ****$p < 0.0001$ and ns, not significant [Two-tailed unpaired Student's *t* test].

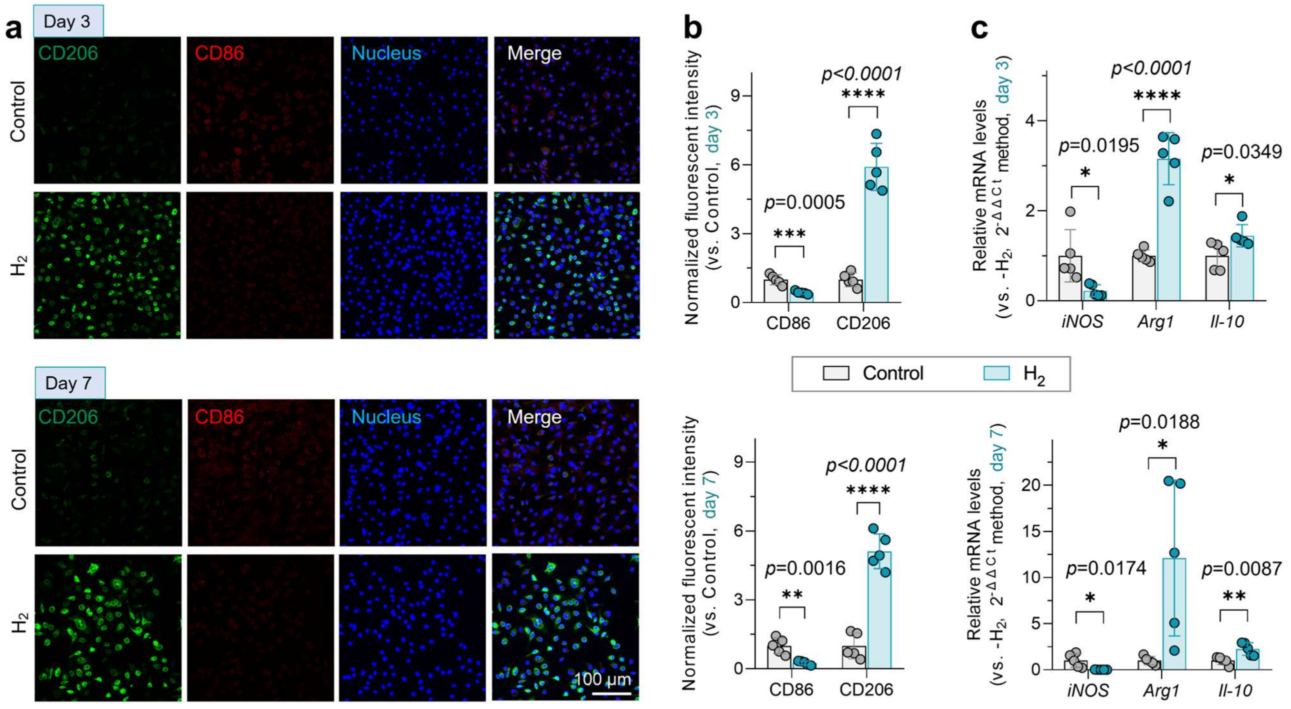

Supplementary Figure 7. Characterization of macrophage polarization after 3 and 7 days of culture in the H_2 incubator or in the general incubator without H_2 (control). (**a**) Immunofluorescence staining, and (**b**) corresponding quantitative analysis of CD86 (M1 marker) and CD206 (M2 marker) expressions ($n = 5$, biologically independent samples). (**c**) mRNA expressions of the M1 marker *iNOS* and the M2 markers *Arg1* and *Il-10* ($n = 5$, biologically independent samples). Data are means ± SD. *$p < 0.05$, **$p < 0.01$, ***$p < 0.001$, and ****$p < 0.0001$ [Two-tailed unpaired Student's t test].

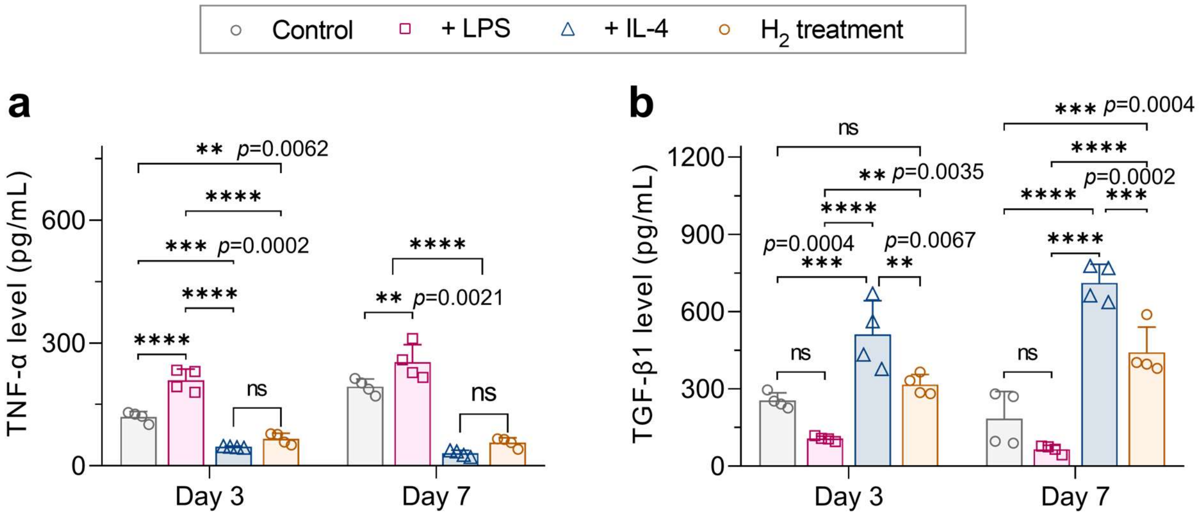

Supplementary Figure 8. The expression of TNF-α (M1 marker, **a**) and TGF-β (M2 marker, **b**) in macrophages after 3 and 7-day treatment with H_2 ($n = 4$, biologically independent samples). Culture in the general incubator was set as the control. M1 and M2 polarizations were induced with 100 ng mL^{-1} LPS and 10 ng mL^{-1} IL-4 in the general incubator, respectively. Data are means ± SD. *$p < 0.05$, **$p < 0.01$, ***$p < 0.001$, ****$p < 0.0001$, and ns, not significant [Two-way ANOVA with Tukey's post hoc test].

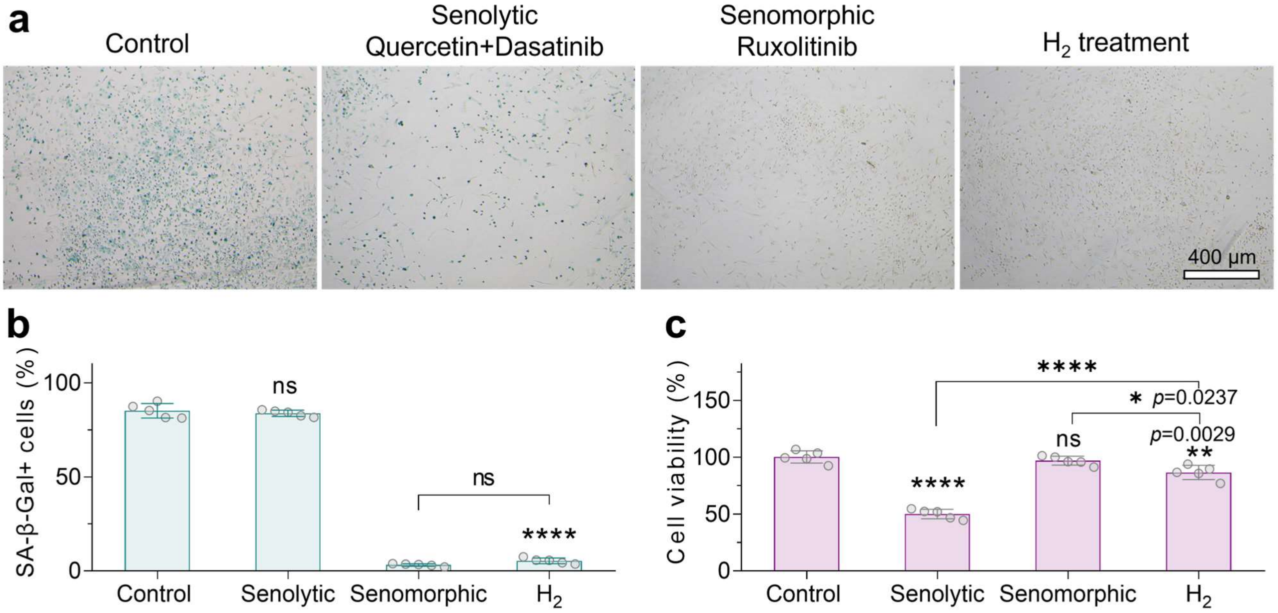

Supplementary Figure 9. The effect of H_2 on senescent osteocytes harvested from aging bone from 24-month-old mice after 3 days of treatment. Representative images of (**a**) SA-β-Gal and the corresponding quantification data (**b**), and the evaluation of cell viability by CCK8 method (**c**). Quercetin (5 μM) plus dasatinib (1 μM) and ruxolitinib (1 μM) were used for positive controls of senolytic and senomorphic drugs, respectively (n = 5, biologically independent samples). Data are means ± SD. *$p < 0.05$, **$p < 0.01$, ****$p < 0.0001$, and ns, not significant [One-way ANOVA with Tukey's post hoc test].

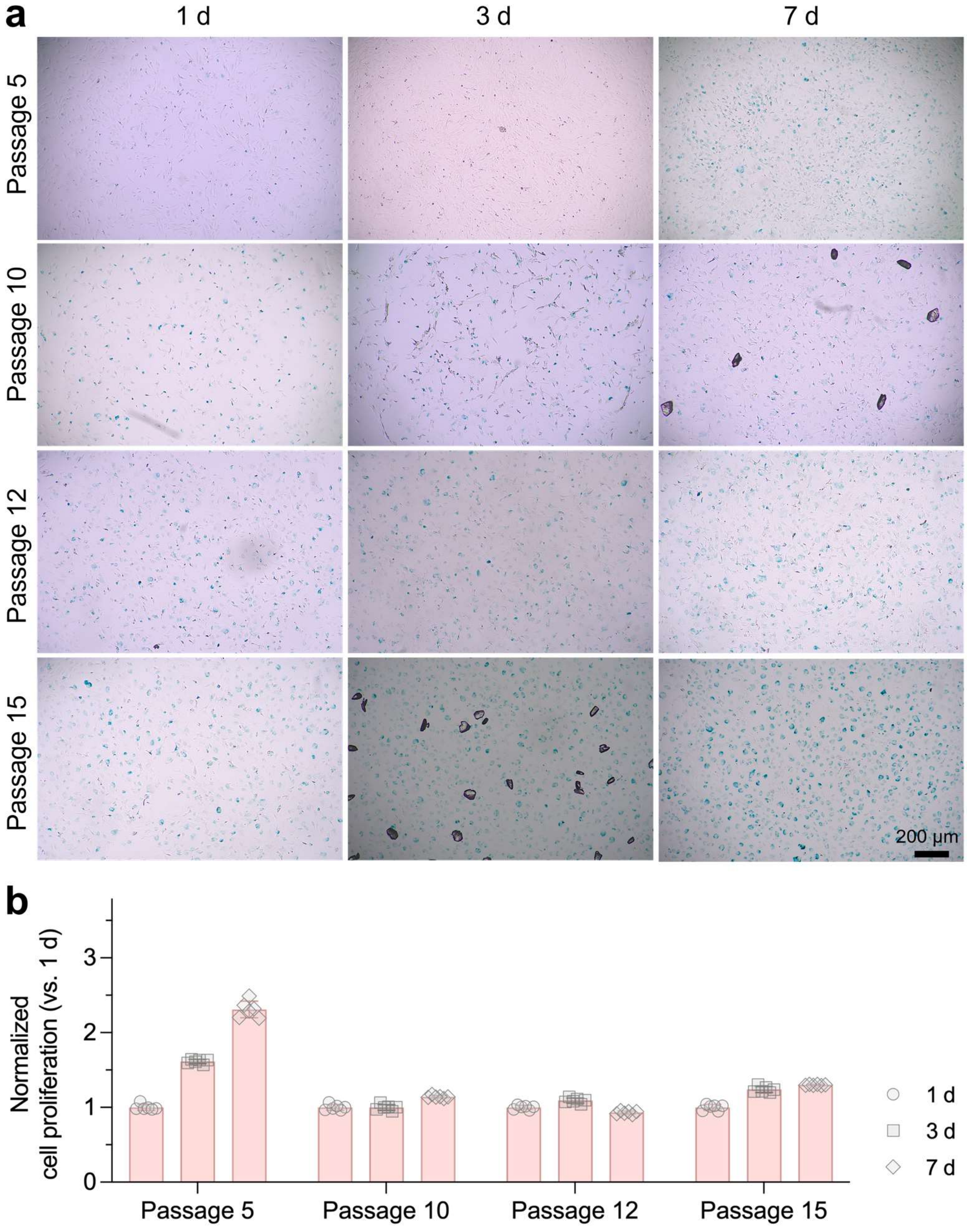

Supplementary Figure 10. SA-β-gal staining (**a**), and proliferation (**b**) of BMSCs (from 6-week-old mice) after 1, 3 and 7 days of incubation at passage 5, 10, 12, and 15 ($n = 6$, biologically independent samples). Data are means ± SD. SA-β-gal staining revealed that BMSCs gradually acquired an increasing senescence phenotype with the increase of passage number. Moreover, the proliferation of BMSCs was arrested at later passages. To evaluate the impact of H_2 on the regenerative functions of senescent stem cells, a replicative senescence model of stem cells was adopted in this study. Since cells will enter a state of replicative senescence after a certain number of divisions, replicative senescent cells have been widely used as a common senescence model for *in vitro* senescence study (Nature 2019, 566, 73; Nature Communications 2019, 10, 5576). Compared with the use of 24-month-old mice, the replicative senescence model has some advantages including higher controllability to senescence degree, higher comparability among different senescence degrees of cells, and more facilitation for application. Therefore, stem cells were extracted from 6-week-old mice, then hastened towards senescence by repeated replication, and finally used for *in vitro* analysis of their regenerative functions in this work.

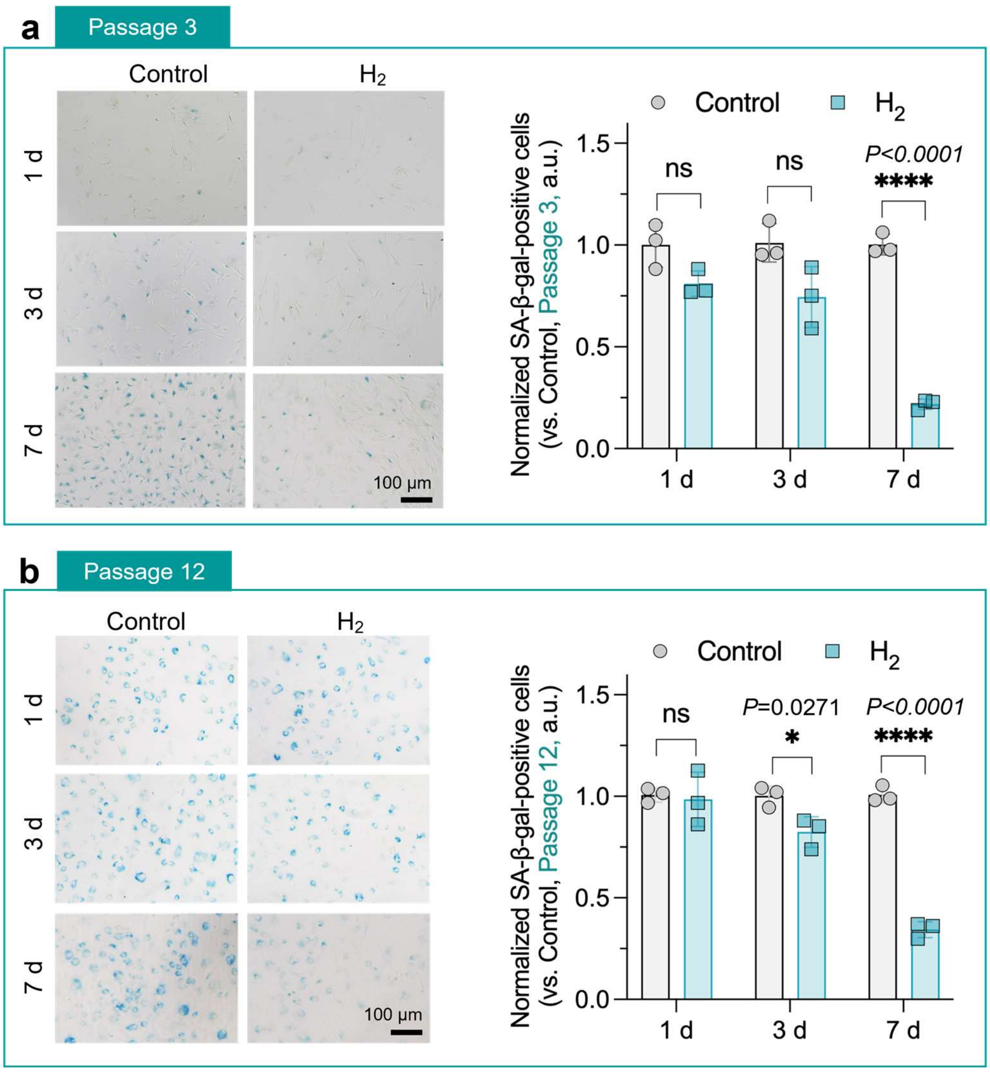

Supplementary Figure 11. SA-β-gal staining and corresponding quantitative analyses of BMSCs (from 6-week-old mice) after 1, 3 and 7 days of treatment at passages 3 (**a**) and 12 (**b**) in the H_2 incubator or in the general incubator without H_2 (n = 3, biologically independent samples). In the control, the SA-β-gal-positive cells increased in number with time both at the early passage and at the late passage. Notably, relative to cells at the early passage, cells at late passage had significant morphological changes with characteristically enlarged and flattened appearance. By contrast, H_2 treatment markedly prevented the progress of cellular senescence, as revealed by significantly less SA-β-gal-positive cells, particularly after 7 days of treatment. Data are means ± SD. $*p < 0.05$, $****p < 0.0001$, and ns, not significant [Two-tailed unpaired Student's t test].

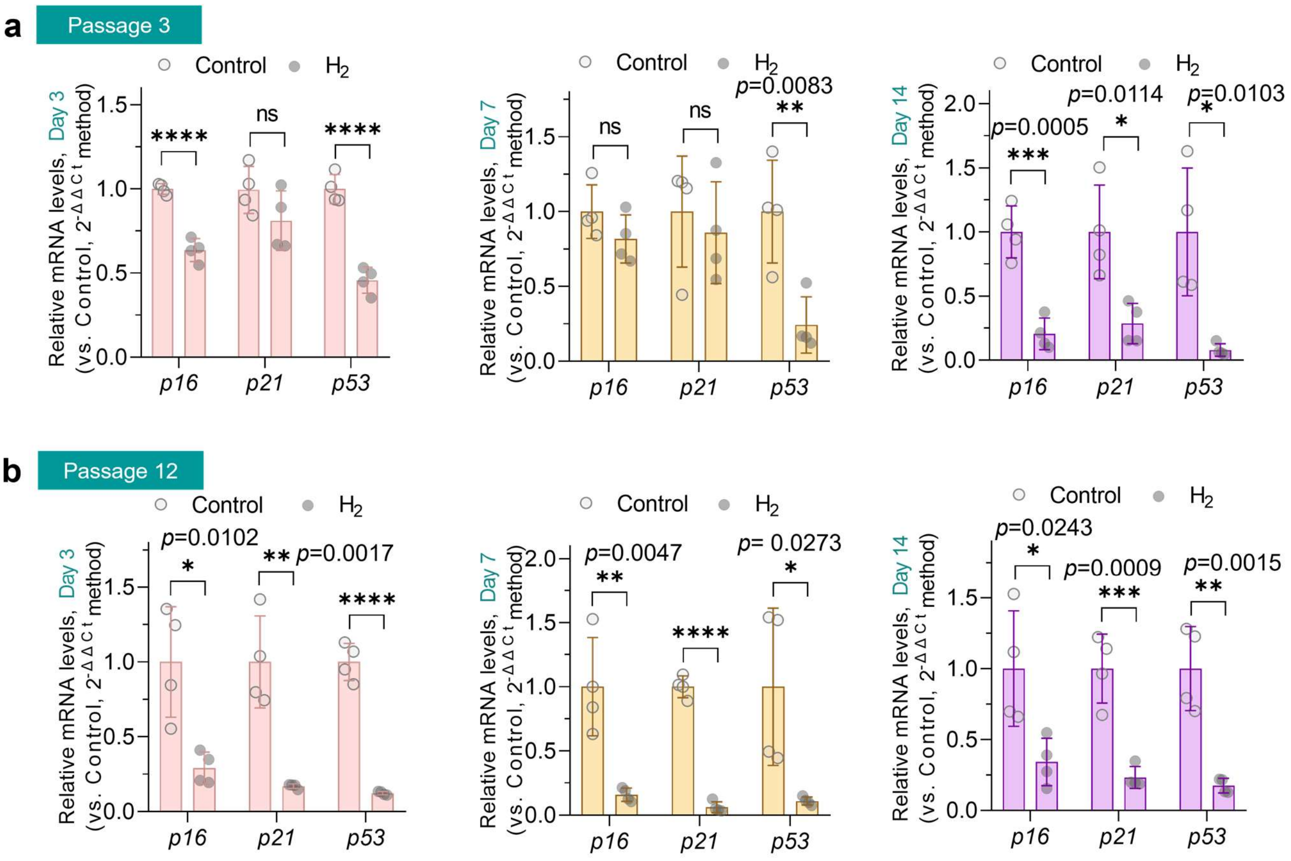

Supplementary Figure 12. mRNA expression of the senescent markers (*p16*, *p21*, *p53*) in BMSCs (from 6-week-old mice) after 3, 7 and 14 days of treatment at passages 3 (**a**) and 12 (**b**) in the H_2 incubator or in the general incubator without H_2 ($n = 4$, biologically independent samples). H_2 was able to regulate senescence. Although BMSCs were at a later passage (passage 12), H_2 had significantly lowered the expression of senescence-related genes. Data are means ± SD. $*p < 0.05$, $**p < 0.01$, $***p < 0.001$, $****p < 0.0001$, and ns, not significant [Two-tailed unpaired Student's *t* test].

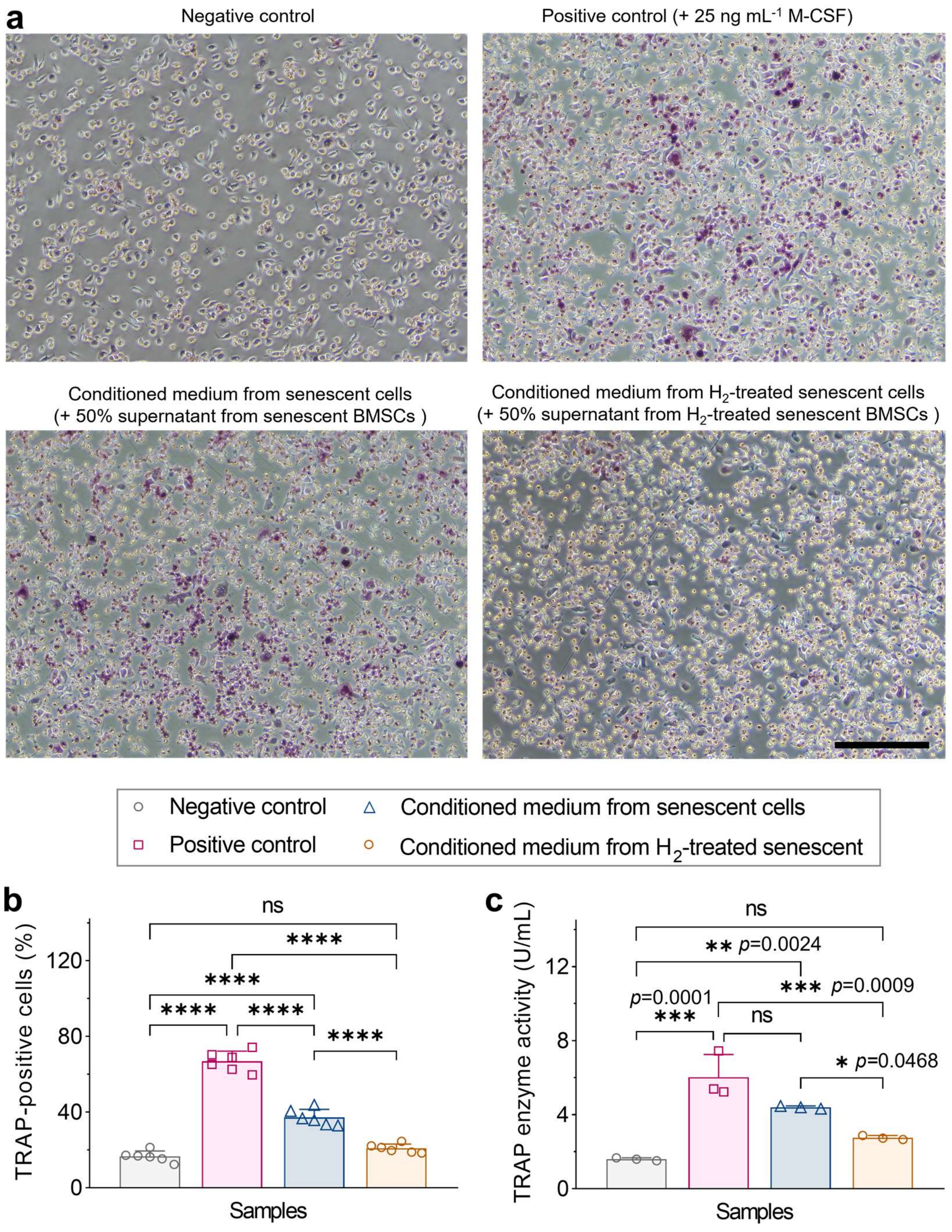

Supplementary Figure 13. H_2 decreases the effect of senescence on osteoclastogenesis *in vitro* by inhibiting the osteoclast differentiation of monocyte osteoclast progenitors. (**a**) Representative images of tartrate-resistant acid phosphatase (TRAP) staining (scale bar, 200 μm), and (**b**) corresponding statistical analyses (n = 6, biologically independent samples). (**c**) TRAP enzyme activity assay (n = 3, biologically independent samples). Data are means ± SD. *$p < 0.05$, **$p < 0.01$, ***$p < 0.001$, ****$p < 0.0001$, and ns, not significant [One-way ANOVA with Tukey's post hoc test].

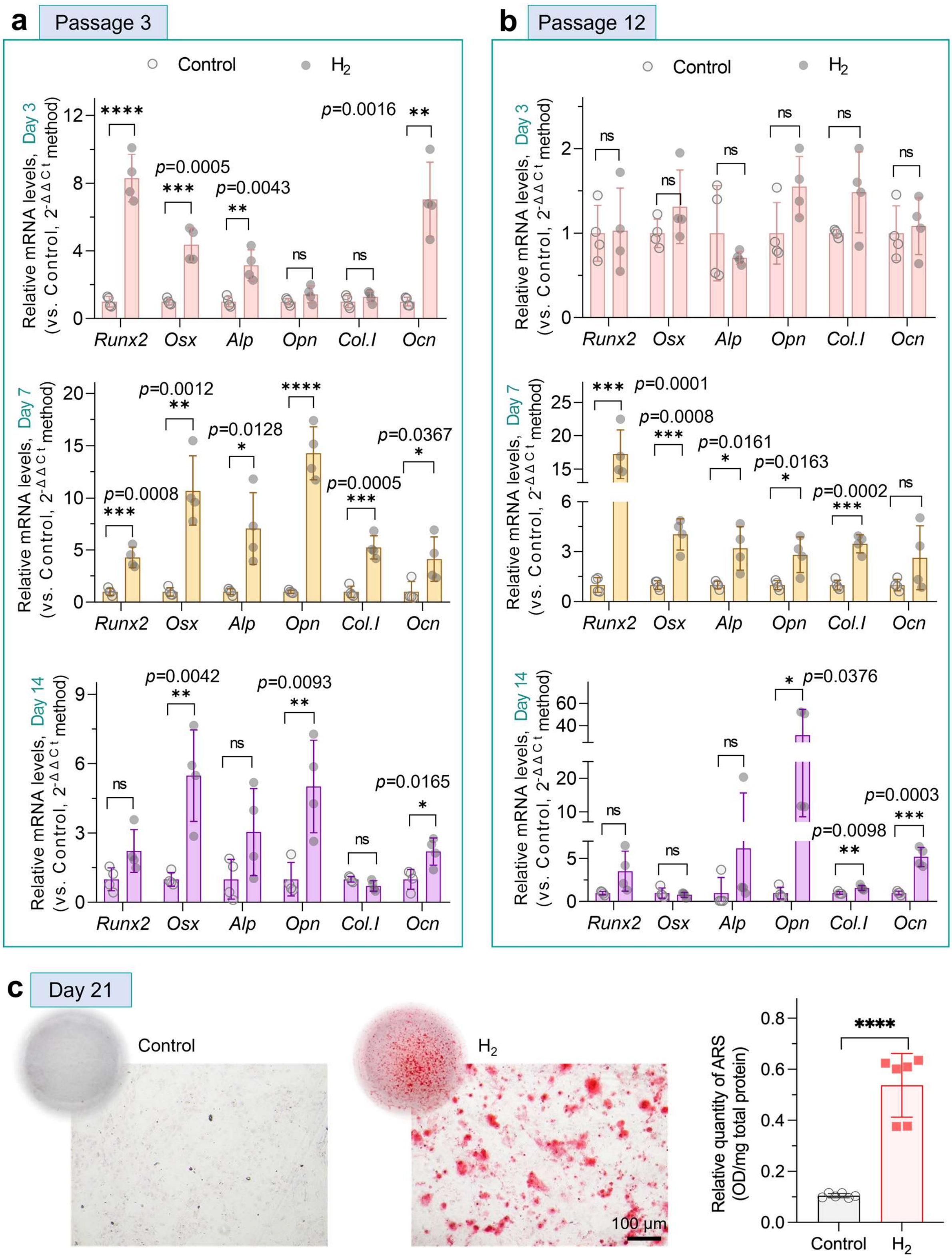

Supplementary Figure 14. Differentiation potential of BMSCs (from 6-week-old mice) in the H_2 incubator or in the general incubator without H_2. (**a, b**) The mRNA expressions of osteogenesis related genes (*Runx2*, *Osx*, *Alp*, *Opn*, *Col.I*, *Ocn*) in BMSCs after 3, 7 and 14 days of treatment (n = 4, biologically independent samples). (**c**) Representative images and quantification of Alizarin Red S (ARS) staining in BMSCs after 21 days of treatment (n = 6, biologically independent samples). Compared to control, H_2 treated BMSCs showed significantly enhanced osteogenic differentiation potential as indicated by enhanced expression of osteogenesis-related genes and higher degree of mineralization. Data are means ± SD. $*p < 0.05$, $**p < 0.01$, $***p < 0.001$, $****p < 0.0001$, and ns, not significant [Two-tailed unpaired Student's *t* test].

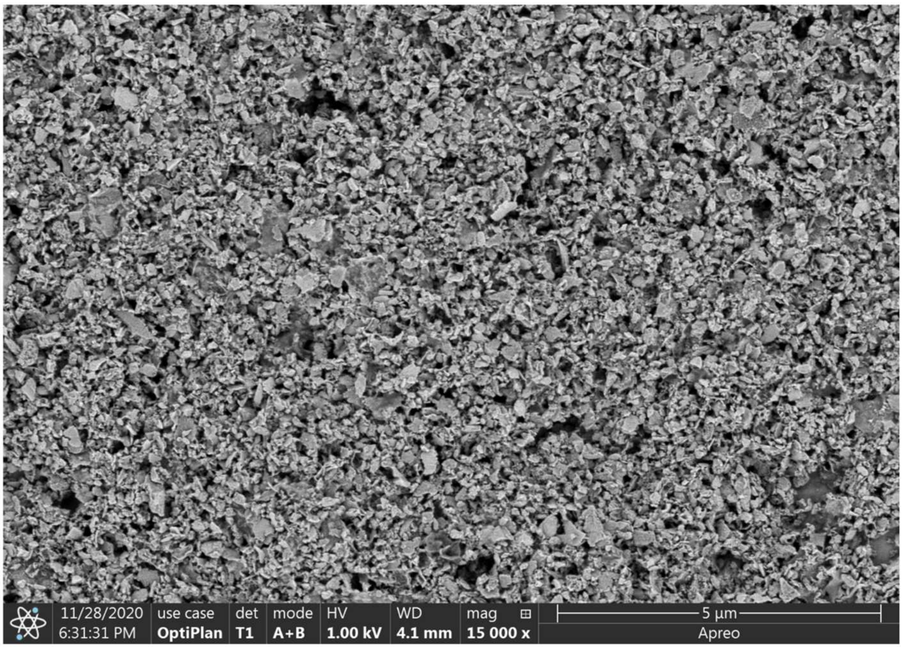

Supplementary Figure 15. SEM image of CSN. The experiments were repeated five times independently with similar results.

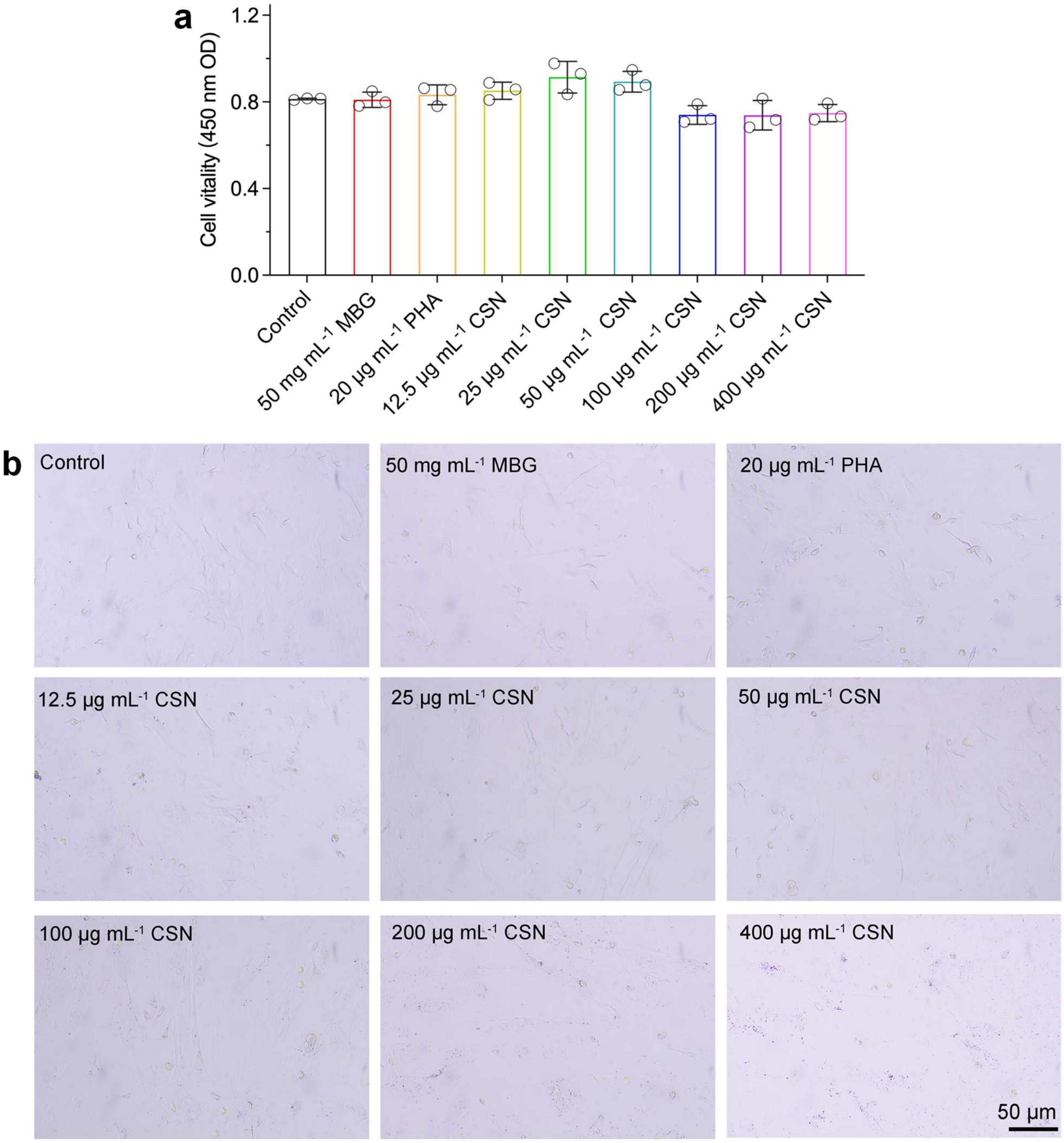

Supplementary Figure 16. Cytocompatibility of CSN. (**a**) Cytotoxicity of CNS at different concentrations against BMSCs (*n* = 3, biologically independent samples). (**b**) Representative images of BMSCs (from 6-week-old mice, passage 3) treated with different concentrations of CSN for 24 h. The experiments were repeated three times independently with similar results. CNS had not caused obvious cytotoxicity against BMSCs in a wide concentration range (0–400 μg mL^{-1}). Data are means ± SD.

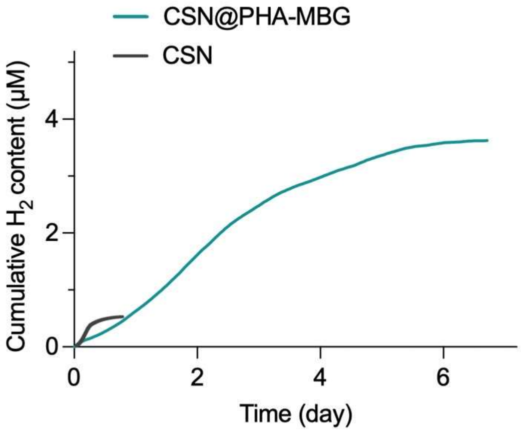

Supplementary Figure 17. Quantification of cumulative H_2 release from CSN@PHA-MBG and CSN in the PBS. Both of CSN@PHA-MBG and CSN contained an equal amount of CSN (1 mg mL^{-1}). Due to low solubility of H_2 in PBS and rapid H_2 release from CSN within 24 h, a large amount of H_2 was spilled from the solution, causing a significant difference in the cumulative amount of H_2 release between CSN@PHA-MBG and CSN.

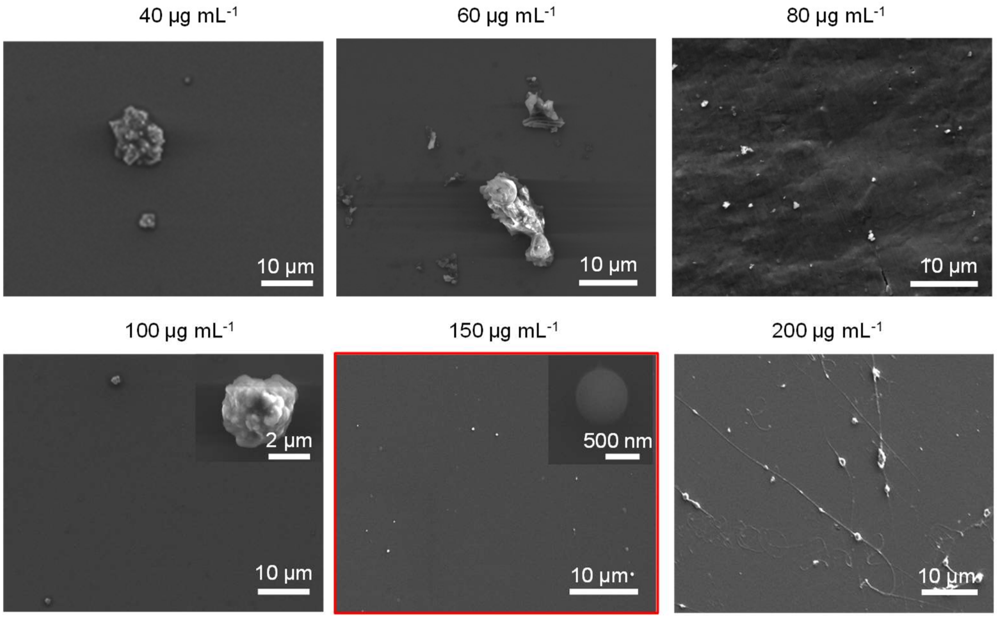

Supplementary Figure 18. SEM images of PHA solutions after electrostatic spraying at different concentrations. For a better morphological outcome, the PHA solution at the concentration of 150 μg mL^{-1} was adopted and applied in this study. The experiments were repeated three times independently with similar results.

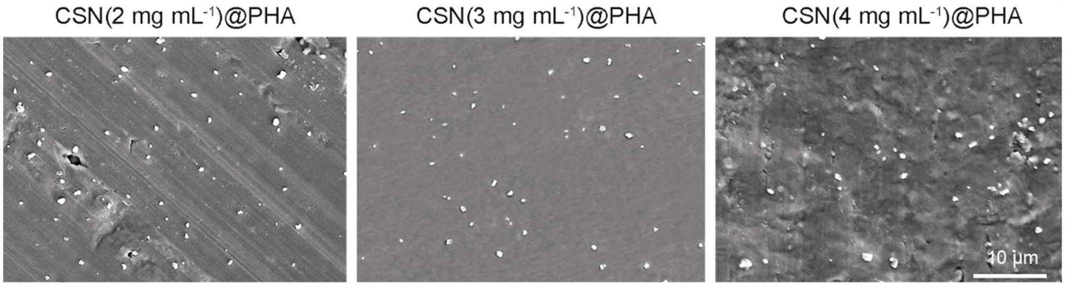

Supplementary Figure 19. SEM images of CNS@PHA after electrostatic spraying with PHA solutions (150 μg mL^{-1}) at different CNS concentrations. The experiments were repeated three times independently with similar results.

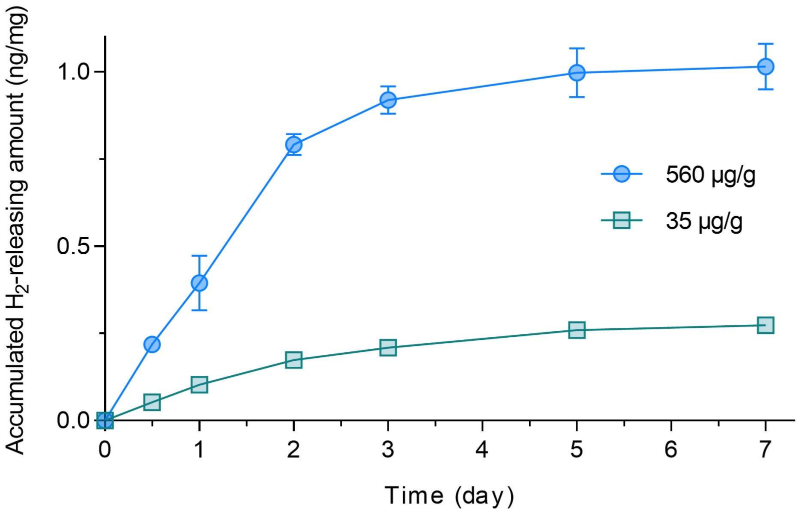

Supplementary Figure 20. Accumulated amount of hydrogen release from CNS@PHA-MBG scaffolds with different CSN contents (35 μg g^{-1} and 560 μg g^{-1}) in the PBS (pH 7.4). n = 3, biologically independent samples. Data are means ± SD.

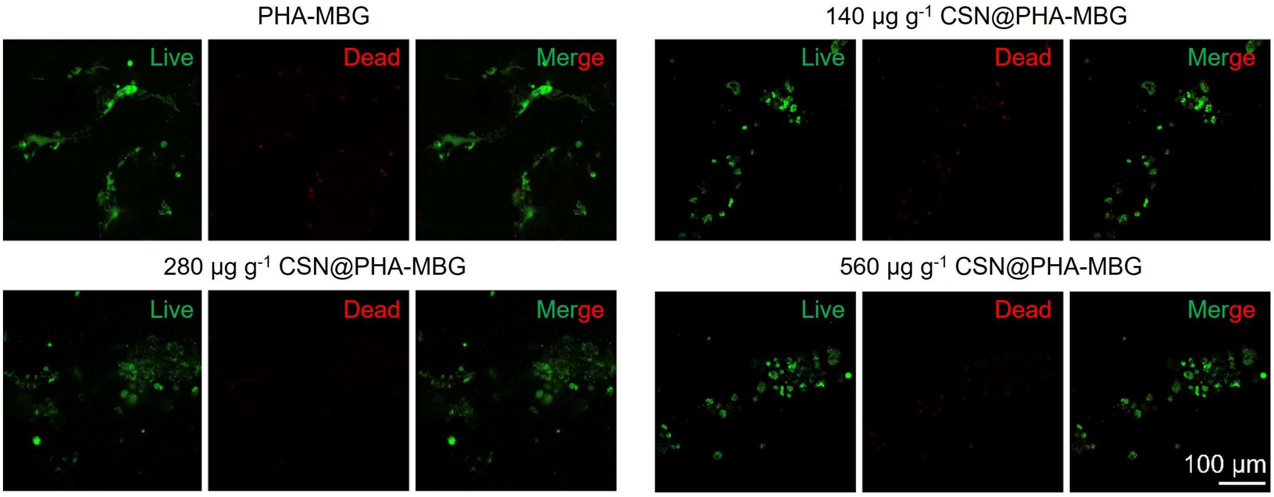

Supplementary Figure 21. Live/Dead fluorescence images of BMSCs seeded on PHA-MBG or CSN@PHA-MBG scaffolds with different CSN contents (140 μg g^{-1}, 280 μg g^{-1}, and 560 μg g^{-1}). The experiments were repeated three times independently with similar results.

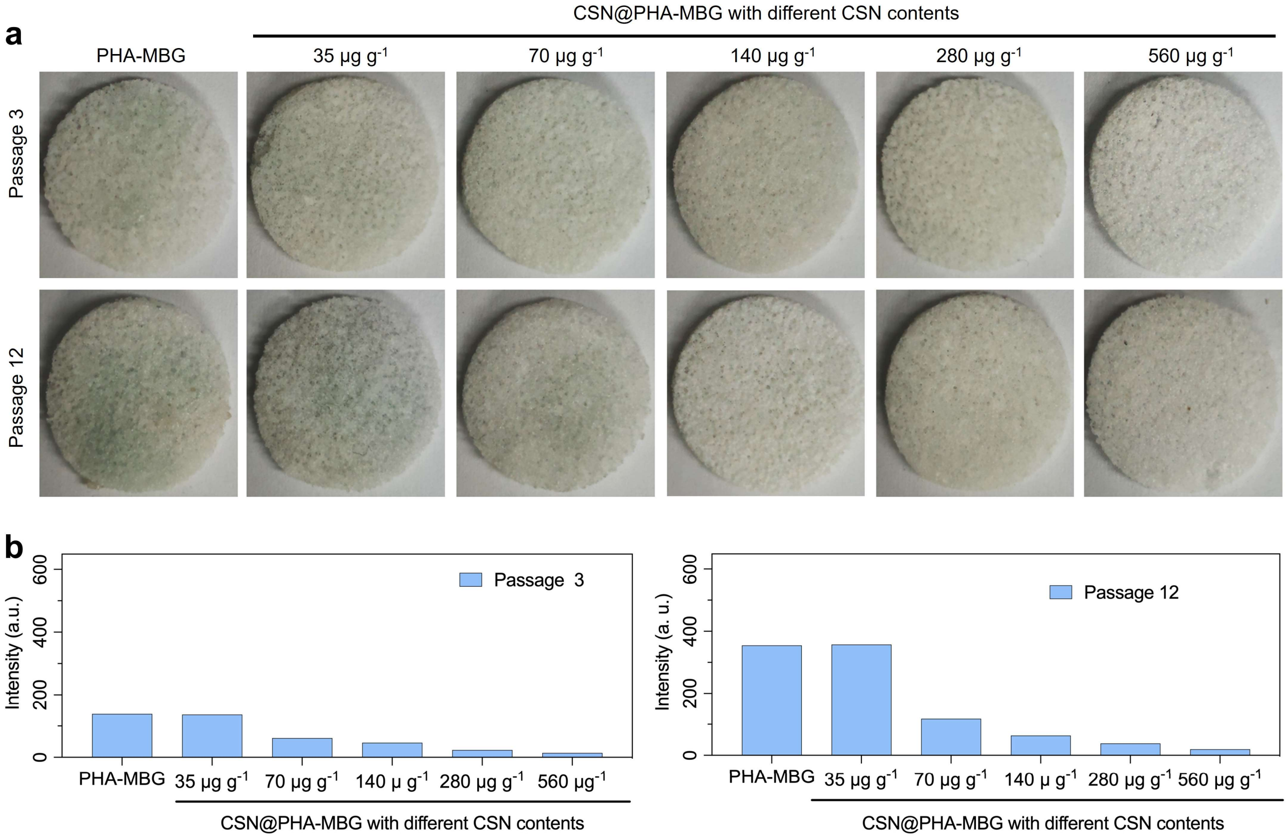

Supplementary Figure 22. SA-β-gal staining (**a**) and corresponding quantitative analyses (**b**) of BMSCs seeded on PHA-MBG or CSN@PHA-MBG scaffolds with different CSN contents (n = 2, biologically independent samples). Data are means.

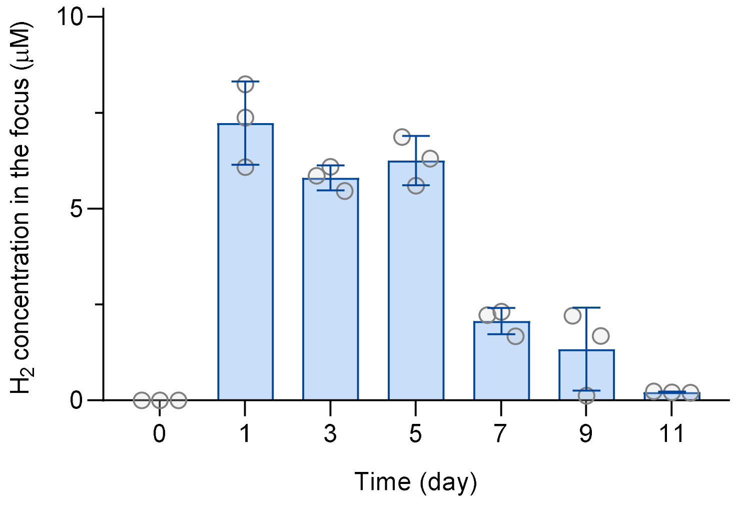

Supplementary Figure 23. H_2 concentration at the site of bone defect where was filled with the CSN@PHA-MBG scaffold for different time durations (n = 3, biologically independent samples). Data are means ± SD.

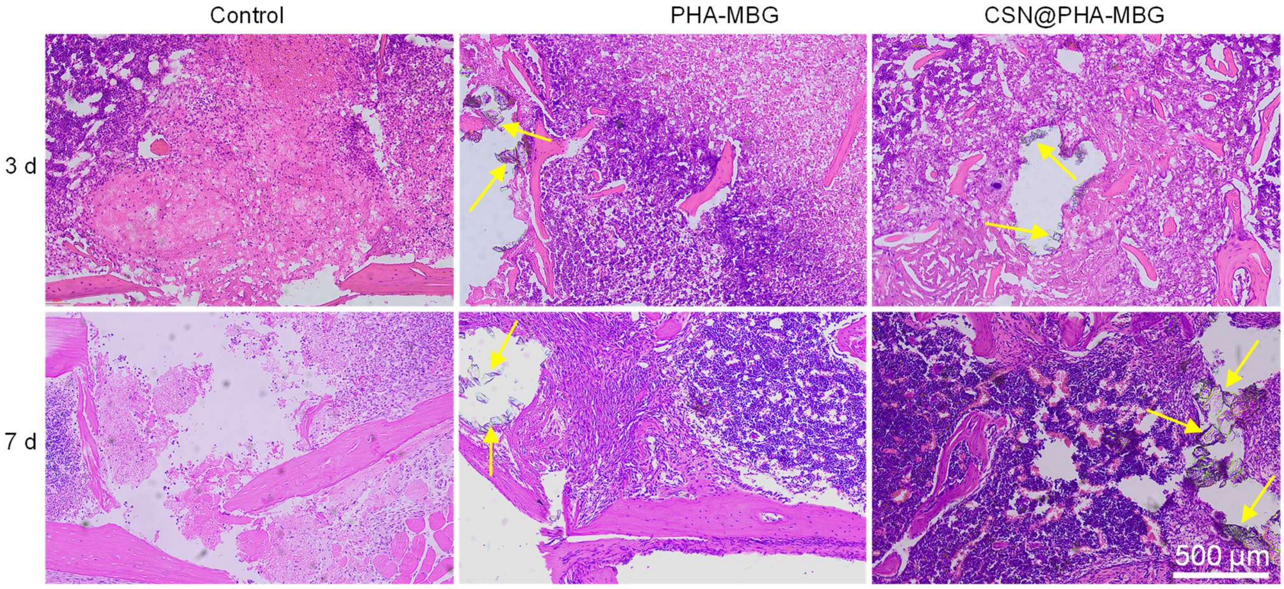

Supplementary Figure 24. HE-stained images of the bone defect sited tissues on day 3 and 7. Yellow arrows indicate the implanted materials. In the control group, the defect site was filled by fibrous tissues at day 3 and unpredictable bone fracture was observed at day 7. In the scaffold groups, neo-tissue was found in the defect site. However, PHA-MBG was wrapped with plentiful fibrous tissues at day 7. This phenomenon was not observed in the CSN@PHA-MBG group.

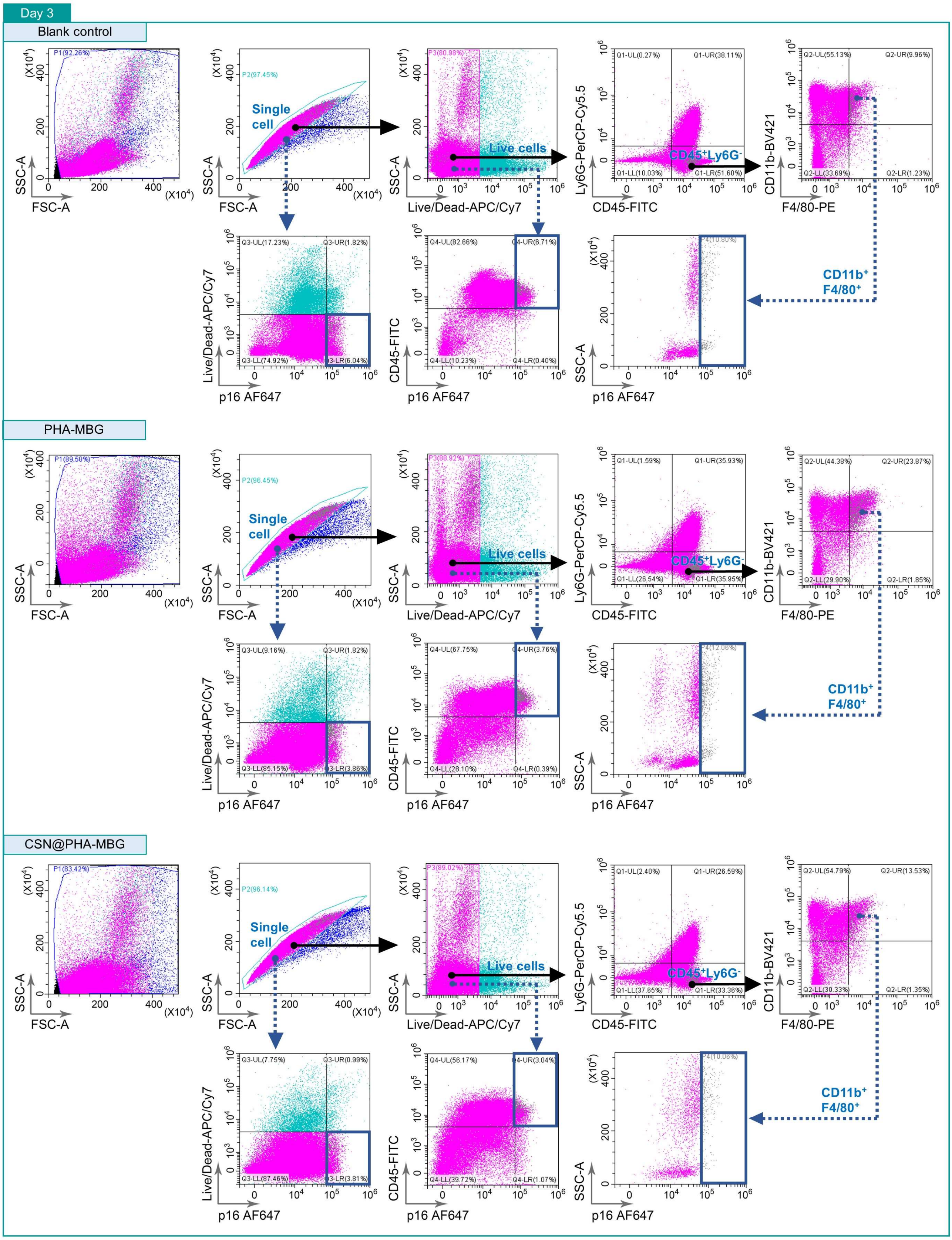

Supplementary Figure 25. Representative gating strategy of flow cytometry for sorting total senescent cells ($p16^+$), senescent myeloid cells ($CD45^+p16^+$) and senescent macrophages ($CD45^+Ly6G^-CD11b^+F4/80^+p16^+$) on day 3.

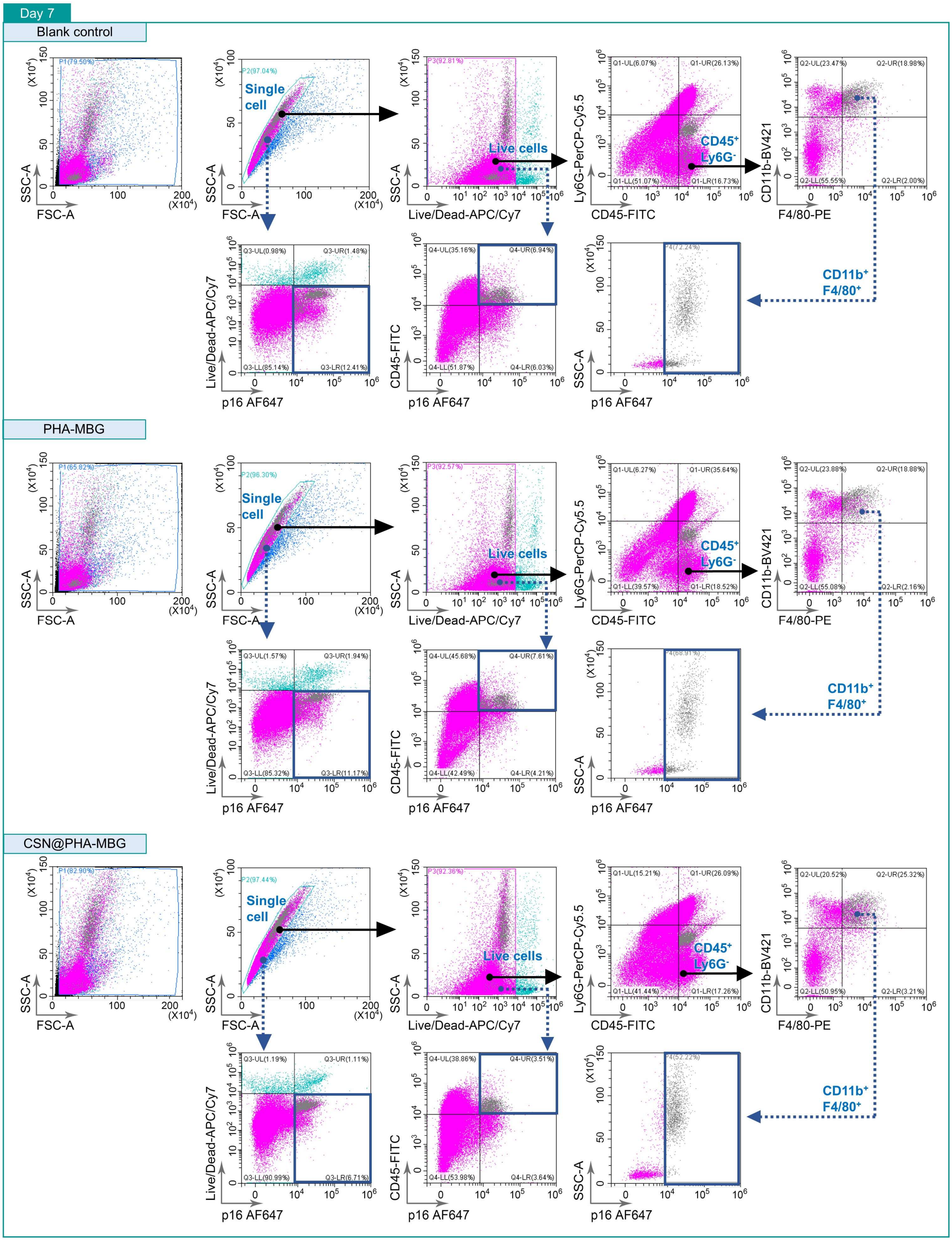

Supplementary Figure 26. Representative gating strategy of flow cytometry for sorting total senescent cells ($p16^+$), senescent myeloid cells ($CD45^+p16^+$) and senescent macrophages ($CD45^+Ly6G^-CD11b^+F4/80^+p16^+$) on day 7.

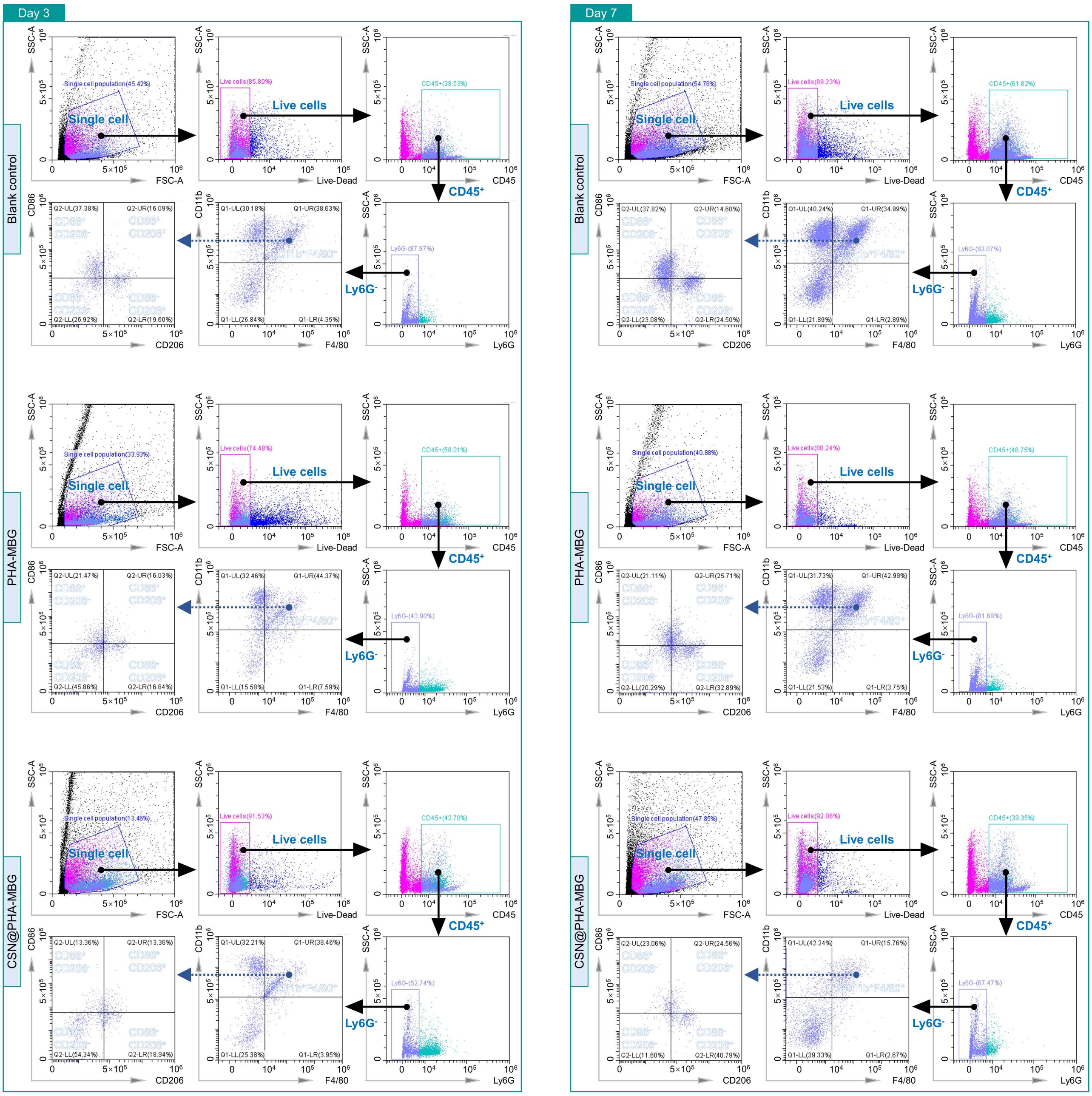

Supplementary Figure 27. Representative gating strategy of flow cytometry for macrophage polarization analyses on day 3 and day 7. The population of M1 macrophages: $CD45^{+}Ly6G^{-}CD11b^{+}F4/80^{+}CD86^{+}CD206^{-}$; the population of M2 macrophages: $CD45^{+}Ly6G^{-}CD11b^{+}F4/80^{+}CD86^{-}CD206^{+}$.

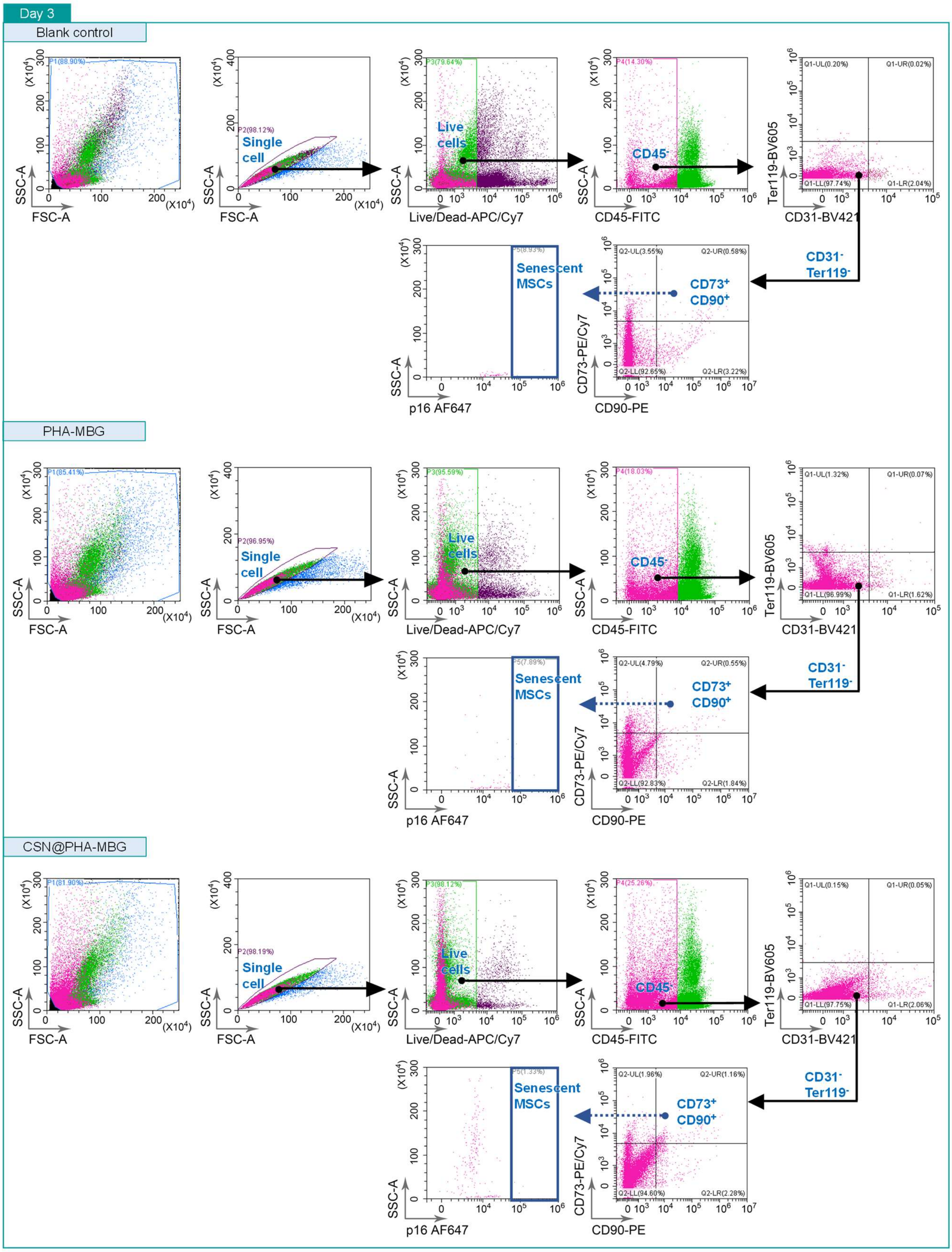

Supplementary Figure 28. Representative gating strategy of flow cytometry for sorting recruited MSCs ($CD45^-Ter119^-CD31^-CD73^+CD90^+$) and senescent MSCs ($CD45^-Ter119^-CD31^-CD73^+CD90^+p16^+$) on day 3.

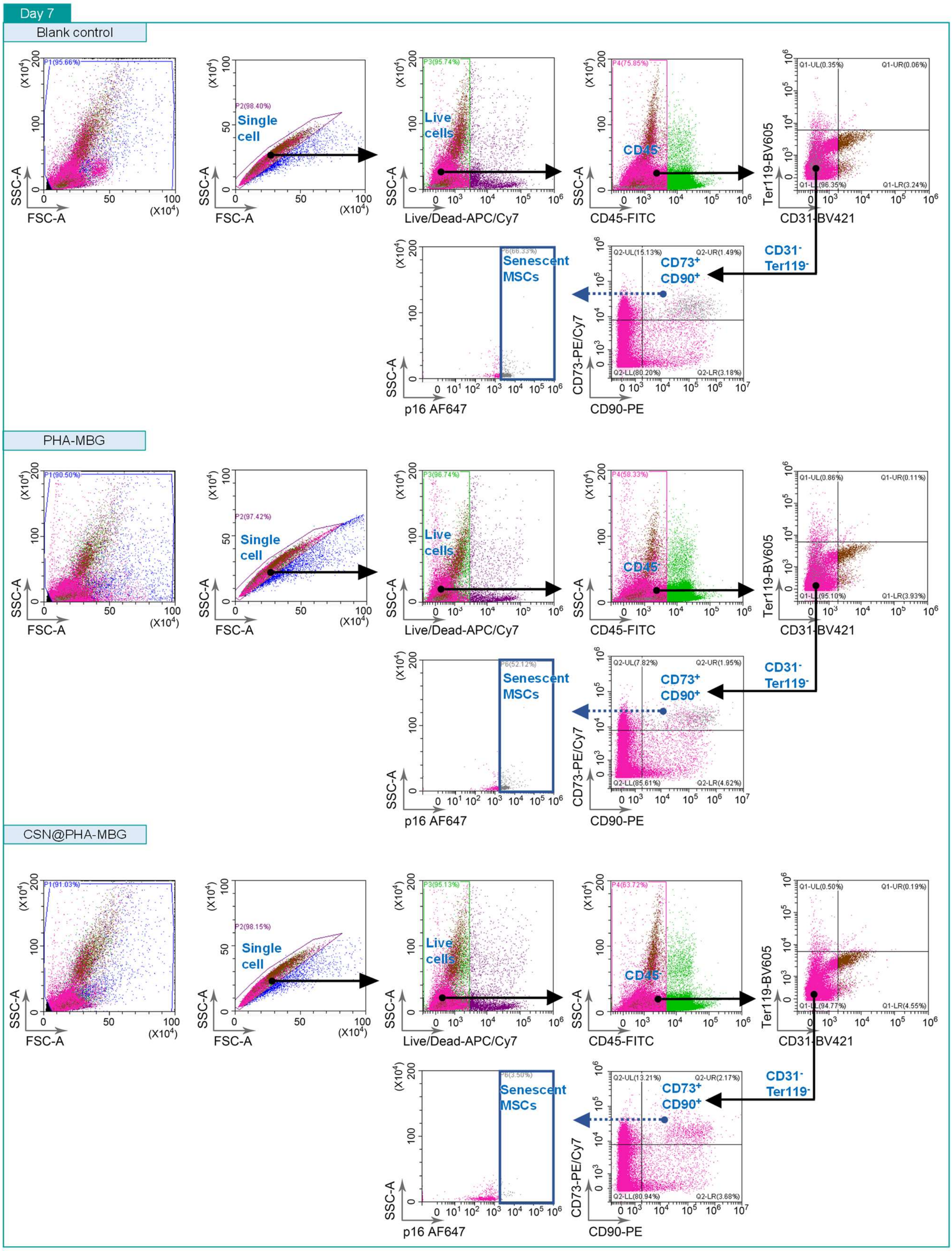

Supplementary Figure 29. Representative gating strategy of flow cytometry for sorting recruited MSCs (CD45$^-$Ter119$^-$CD31$^-$CD73$^+$CD90$^+$) and senescent MSCs (CD45$^-$Ter119$^-$CD31$^-$CD73$^+$CD90$^+$p16$^+$) on day 7.

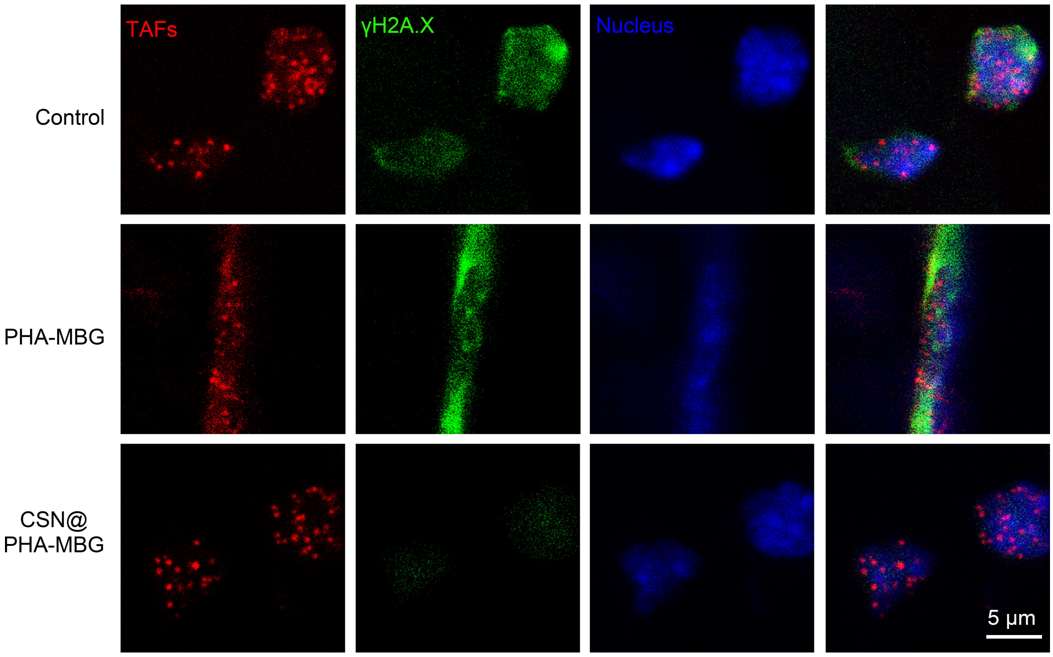

Supplementary Figure 30. Representative images of γ-H2A.X immuno-FISH *in vivo* at day 3. Compared with control and PHA-MBG, CSN@PHA-MBG exhibited less γ-H2A.X expression and fewer enrichment of γ-H2AX at TAFs.

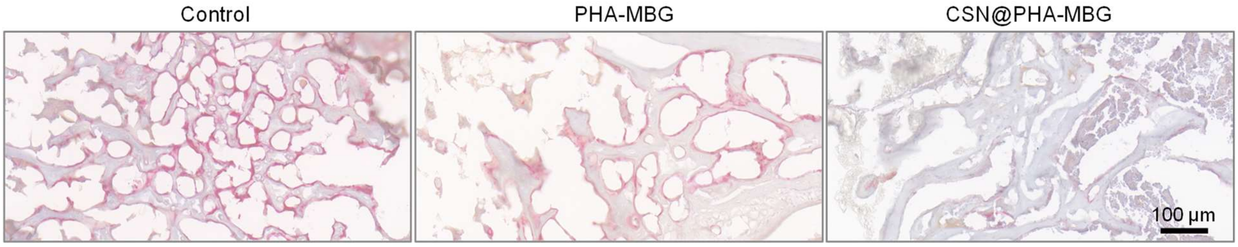

Supplementary Figure 31. Representative images of TRAP-stained osteoclasts at the site of bone defect where was filled with nothing (control), PHA-MBG, or CSN@PHA-MBG scaffold after 28 days of treatment. TRAP staining was finalized with a staining kit according to the manufacturer's instructions (Sigma-Aldrich, 387A). The experiments were repeated three times independently with similar results.

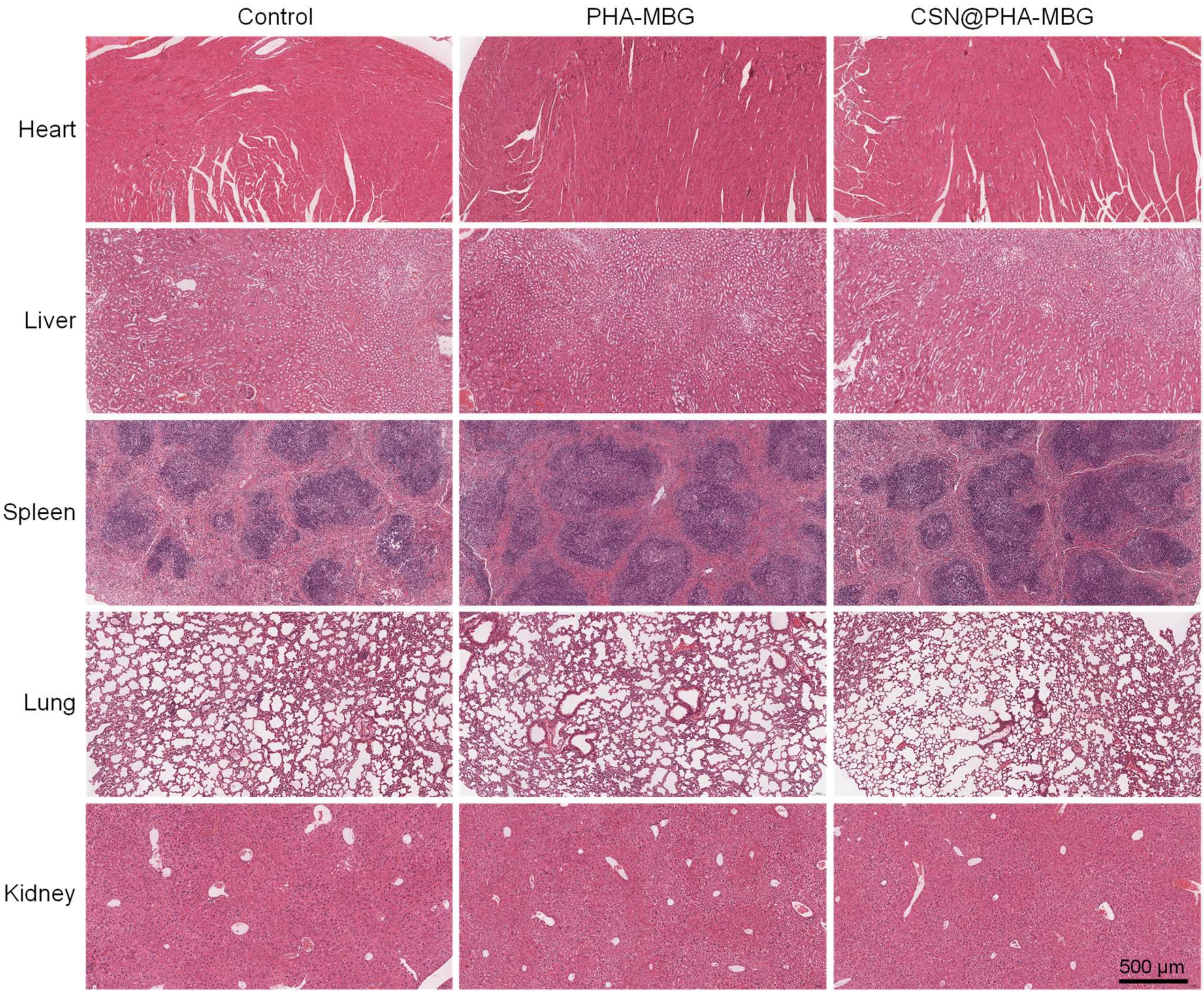

Supplementary Figure 32. Representative HE staining of main organs (heart, liver, spleen, lung and kidney) after 28-day treatment. The experiments were repeated four times independently with similar results.

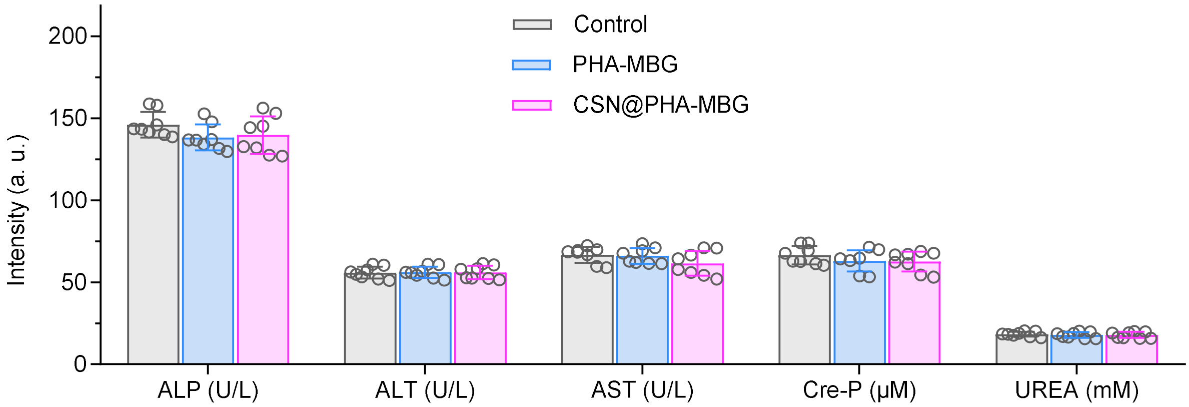

Supplementary Figure 33. Liver and kidney functions after 28-day treatment. ALP, alkaline phosphatase; ALT, alanine transaminase; AST, aspartate transaminase; Cre-P, creatinine; UREA, blood urea. n = 8, biologically independent samples. Data are means ± SD.

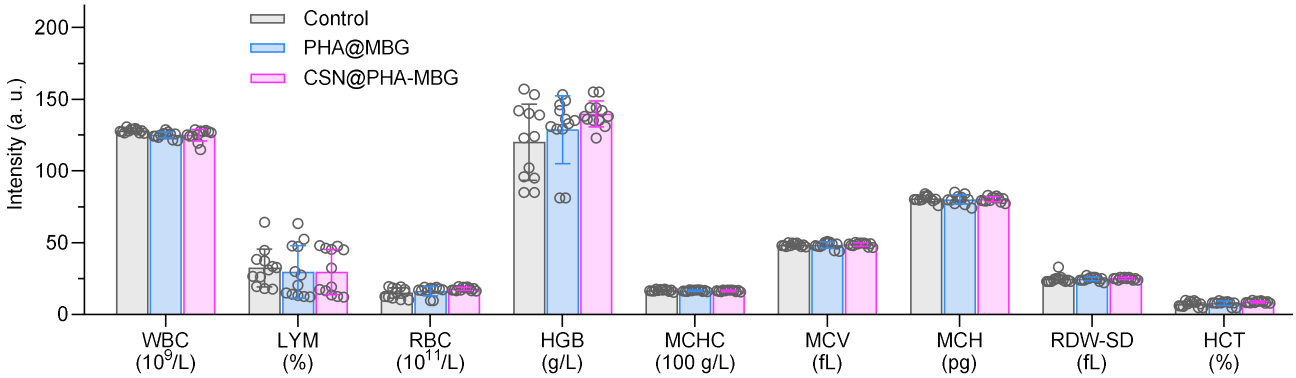

Supplementary Figure 34. The blood routine examination of white blood cells (WBC), lymphocytes percentage (LYM), red blood cells (RBC), hemoglobin (HGB), mean corpuscular hemoglobin concentration (MCHC), mean corpuscular volume (MCV), mean corpuscular hemoglobin (MCH), red blood cell volume distribution width (RDW-SD) and hematocrit (HCT). n = 12, biologically independent samples. Data are means ± SD.

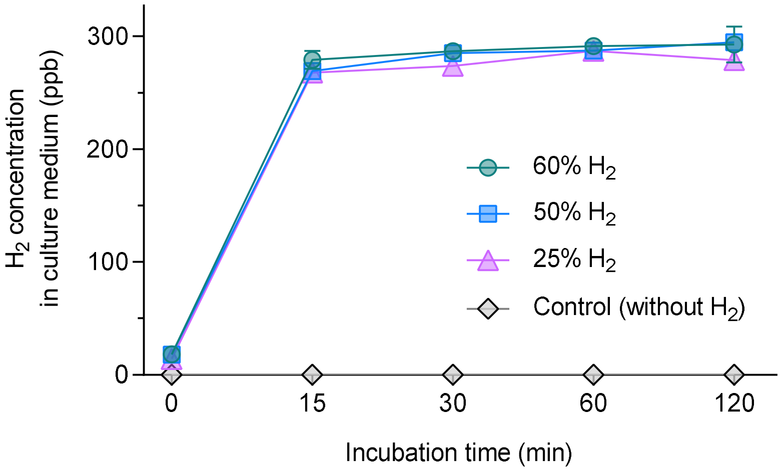

Supplementary Figure 35. H_2 concentration in the culture medium during incubation in the hydrogen incubator with various hydrogen gas contents (25%, 50% and 60%) or in the general incubator without H_2 supplement (control) (n = 3, biologically independent samples). Data are means ± SD.

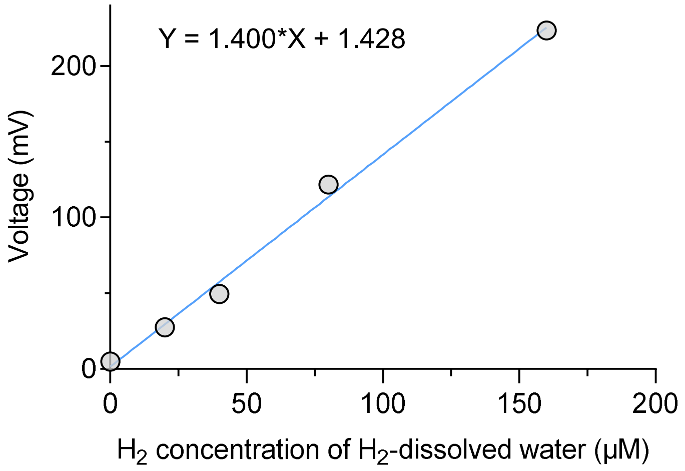

Supplementary Figure 36. The standard curve for measurement of H_2 concentration by microelectrode.

Supplementary Table 1. Primers for qPCR analysis (species, mouse).

**Gene**	**Forward**	**Reverse**
E11/gp38	AACAAGTCACCCCAATAGAGATAAT	CTAACAAGACGCCAACTATGATTC
Sost	TGAGAACAACCAGACCATGAAC	TCAGGAAGCGGGTGTAGTG
iNOS	GCAGAGATTGGAGGCCTTGTG	GGGTTGTTGCTGAACTTCCAGTC
Arg1	CATTGGCTTGCGAGACGTAGAC	GCTGAAGGTCTCTTCCATCACC
Il-10	GCTGTCATCGATTTCTCCCCT	AGATGTCAAACTCATTCATGGCC
p16	GAACTCTTTCGGTCGTACCC	CGAATCTGCACCGTAGTTGA
p21	ATCACCAGGATTGGACATGG	CGGTGTCAGAGTCTAGGGGA
p53	GAGGTTGGCTCTGACTGTACC	TCCGTCCCAGTAGATTACCAC
Runx2	CTCATCCCAGTATGAGAGTAGGTGT	TCTGTAATCTGACTCTGTCCTTGTG
Osterix	CCTACTTACCCATCTGACTTTGCT	CTTATAGACATCTTGGGGTAGGACA
Alp	GGGGACATGCAGTATGAGTT	GGCCTGGTAGTTGTTGTGAG
Opn	CTGCTAGTACACAAGCAGACA	CATGAGAAATTCGGAATTTCAG
Col.1	TGCTTGAAGACCTATGTGGGTA	AAAGGCAGCATTTGGGGTAT
Ocn	TGAACAGACTCCGGCG	GATACCGTAGATGCGTTTG
Gapdh	CCATTCTTCCACCTTTGATGCTG	GTCCAGGGTTTCTTACTCCTTGG
β-actin	CAACCGTGAAAAGATGACCC	GTAGATGGGCACAGTGTGGG
