# Supplementary material for: Local H2 release remodels senescence microenvironment for improved repair of injured bone
Source: Nat Commun. 2023 Nov 27;14:7783. doi: 10.1038/s41467-023-43618-z (PMC10682449; doi:10.1038/s41467-023-43618-z)
Supplement: Supplementary file 3 — Reporting Summary [file 41467_2023_43618_MOESM3_ESM.pdf]

nature portfolio

Corresponding author(s): Qianjun He, Wei Tang, Changsheng Liu

Last updated by author(s): Oct 8, 2023



# Reporting Summary

Nature Portfolio wishes to improve the reproducibility of the work that we publish. This form provides structure for consistency and transparency in reporting. For further information on Nature Portfolio policies, see our Editorial Policies and the Editorial Policy Checklist.

## Statistics

For all statistical analyses, confirm that the following items are present in the figure legend, table legend, main text, or Methods section.

| n/a | Confirmed | |
|---|---|---|
| ☐ | ☒ | The exact sample size (*n*) for each experimental group/condition, given as a discrete number and unit of measurement |
| ☐ | ☒ | A statement on whether measurements were taken from distinct samples or whether the same sample was measured repeatedly |
| ☐ | ☒ | The statistical test(s) used AND whether they are one- or two-sided<br>*Only common tests should be described solely by name; describe more complex techniques in the Methods section.* |
| ☒ | ☐ | A description of all covariates tested |
| ☒ | ☐ | A description of any assumptions or corrections, such as tests of normality and adjustment for multiple comparisons |
| ☐ | ☒ | A full description of the statistical parameters including central tendency (e.g. means) or other basic estimates (e.g. regression coefficient) AND variation (e.g. standard deviation) or associated estimates of uncertainty (e.g. confidence intervals) |
| ☐ | ☒ | For null hypothesis testing, the test statistic (e.g. *F*, *t*, *r*) with confidence intervals, effect sizes, degrees of freedom and *P* value noted<br>*Give P values as exact values whenever suitable.* |
| ☒ | ☐ | For Bayesian analysis, information on the choice of priors and Markov chain Monte Carlo settings |
| ☒ | ☐ | For hierarchical and complex designs, identification of the appropriate level for tests and full reporting of outcomes |
| ☒ | ☐ | Estimates of effect sizes (e.g. Cohen's *d*, Pearson's *r*), indicating how they were calculated |

*Our web collection on statistics for biologists contains articles on many of the points above.*

## Software and code

Policy information about availability of computer code

| | |
|---|---|
| Data collection | XRD data were collected via MiniFlex600.<br>Flow cytometry data were collected from CytoFLEX-S cytometer.<br>Absorbance data were collected from microplate reader on Tecan infinite 2000 PRO.<br>Immunofluorescence data were collected from ZEISS-LSM 880 and LEICA - SP5 Ⅱ.<br>Hydrogen release data were collected from hydrogen electrode (Unisense, Denmark) or GC (Agilent Technologies, 7890B).<br>SEM data were collected from Thermo APREO S.<br>SRμCT data were collected from BL13HB beamline of the Shanghai Synchrotron Radiation Facility and a 2048×2048 sCMOS detector (Flash 4.0, Hamamatsu City, Japan).<br>Biochemical measurement data were collected from cell analyzer (BC-31S, Mindray) and biochemical analyzer (iMagic-M7). |
| Data analysis | Microscopic images were analyzed by using ImageJ (Version 1.52k).<br>Flow cytometry data were analyzed via CytExpert (version 2.3).<br>Statistical data analyses were performed using GraphPad Prism 8 (Version 9) and Origin (version 2021).<br>SRμCT data were analyses by Avizo software and Brucker software. |

For manuscripts utilizing custom algorithms or software that are central to the research but not yet described in published literature, software must be made available to editors and reviewers. We strongly encourage code deposition in a community repository (e.g. GitHub). See the Nature Portfolio guidelines for submitting code & software for further information.

# Data

Policy information about availability of data

All manuscripts must include a data availability statement. This statement should provide the following information, where applicable:

- Accession codes, unique identifiers, or web links for publicly available datasets
- A description of any restrictions on data availability
- For clinical datasets or third party data, please ensure that the statement adheres to our policy

All the data supporting the findings of this study are available in the article and its supplementary information files and from the corresponding author upon reasonable request.

## Research involving human participants, their data, or biological material

Policy information about studies with human participants or human data. See also policy information about sex, gender (identity/presentation), and sexual orientation and race, ethnicity and racism.

| | |
|---|---|
| Reporting on sex and gender | n/a |
| Reporting on race, ethnicity, or other socially relevant groupings | n/a |
| Population characteristics | n/a |
| Recruitment | n/a |
| Ethics oversight | n/a |

Note that full information on the approval of the study protocol must also be provided in the manuscript.

# Field-specific reporting

Please select the one below that is the best fit for your research. If you are not sure, read the appropriate sections before making your selection.

[x] Life sciences [ ] Behavioural & social sciences [ ] Ecological, evolutionary & environmental sciences

For a reference copy of the document with all sections, see nature.com/documents/nr-reporting-summary-flat.pdf

# Life sciences study design

All studies must disclose on these points even when the disclosure is negative.

| | |
|---|---|
| Sample size | n = 8 biologically independent samples for ELISA measurement for Figure 1b,<br>n = 4 biologically independent samples for Figure 3,<br>n = 2 biologically independent samples for Figure 4c<br>n = 5 biologically independent samples for Figure 4d,<br>n = 4 biologically independent samples for Figure 5,<br>n = 8 biologically independent samples for Figure 6b<br>n = 3 biologically independent samples for Figure 6e,f,g, n=4 for Figure 6i<br>For all experiments, the sample size was determined according to the experimental conditions. |
| Data exclusions | No data were excluded. |
| Replication | We confirm that all attempts at replication were successful in a month. |
| Randomization | For in vivo and in vitro studies, samples were randomly allocated to different experimental groups. |
| Blinding | Both in vitro cells and in vivo mice allocations were conducted in a blinded manner. |

# Reporting for specific materials, systems and methods

We require information from authors about some types of materials, experimental systems and methods used in many studies. Here, indicate whether each material, system or method listed is relevant to your study. If you are not sure if a list item applies to your research, read the appropriate section before selecting a response.

## Materials & experimental systems

| n/a | Involved in the study | |
|---|---|---|
| | ☒ | Antibodies |
| | ☒ | Eukaryotic cell lines |
| ☒ | | Palaeontology and archaeology |
| | ☒ | Animals and other organisms |
| ☒ | | Clinical data |
| ☒ | | Dual use research of concern |
| ☒ | | Plants |

## Methods

| n/a | Involved in the study | |
|---|---|---|
| ☒ | | ChIP-seq |
| | ☒ | Flow cytometry |
| ☒ | | MRI-based neuroimaging |

# Antibodies

Antibodies used

For in vitro immunofluorescence studies, following antibodies were used:
Primary antibodies: HO-1 (Abcam, ab68477), p16INK4A (Abcam, ab211542), Ki67 (Novus, NB500-170), anti-p21 (Abcam, ab188224), γH2A.X (CST, 9718T), SOST (Bioss, bs-10200R), CD86 (Sant Cruz, sc-28347), CD206 (CST, 24595S). Secondary antibodies: Alexa Fluor 647 conjugated goat anti-mouse IgG H&L (Abcam, ab150115) and Alexa Fluor 488 conjugated goat anti-rabbit IgG H&L (Abcam, ab150081).

For in vivo immunofluorescence studies, following antibodies were used:
Primary antibodies: F4/80 (Abcam, ab6640), iNOS (Abcam, ab15323), Arg1 (NOVUS Biologicals, NBP2-03618), p16INK4A (CST, 29271S), Leptin R (R&D system, BAF497), CD31 (Abcam, ab28364), Endomucin (Santa Cruz, sc-65495), Osterix (Santa Cruz, sc-22536-R), Vpp3 (Abcam, ab200839). Secondary antibodies: Alexa Fluor 555 conjugated goat anti-rabbit IgG H&L (Abcam, ab150086), Alexa Fluor 555 conjugated streptavidin (Thermo, S32355), Alexa Fluor 488 conjugated goat anti-rat IgG H&L (Jackson ImmunoResearch, 112-545-062) and Alexa Fluor 647 conjugated goat anti-mouse IgG H&L (Jackson ImmunoResearch, 115-605-003).

For flow-cytometry studies, following antibodies were used:
anti-mouse CD16/32 antibody (Biolegend, 101320), FITC-conjugated CD45 (BD, 553079), PerCP-Cy5.5 conjugated Ly6G (BD, 560602), BV421 conjugated CD11b (BD, 562605), PE conjugated F4/80 (BD, 565410), PE-Cy7 conjugated CD86 (BD, 560582), Alexa Fluor 647 conjugated CD206 antibody (BD, 565250), BV421 conjugated CD31 (Biolegend, 102424), BV605 conjugated Ter119 (Biolegend, 116239), PE/Cyanine7 conjugated CD73 (Biolegend, 127224), PE conjugated CD90 (BD, 553006), anti-p16INK4a antibody(Abcam, ab211542), and Alexa Fluor 647 conjugated donkey anti-Rabbit IgG H&L (Abcam, ab150075).

Validation

All primary antibodies have been validated for the species and application by the manufacturer's website and relevant references.

# Eukaryotic cell lines

Policy information about cell lines and Sex and Gender in Research

Cell line source(s)

All primary cells were extracted from old C57BL/6J male mice (24 months) or young C57BL/6J male mice (6 weeks). Mesenchymal stem cells were extracted from fresh bone marrow. Macrophages were differentiated from monocytes isolated from fresh bone marrow. Osteocytes were isolated from fresh femur and tibia.

Authentication

Osteocytes were authenticated by the specific marker of sclerostin and E11/gp38.

Mycoplasma contamination

No mycoplasma contamination was detected.

Commonly misidentified lines
(See ICLAC register)

No commonly misidentified cell lines were used.

# Animals and other research organisms

Policy information about studies involving animals; ARRIVE guidelines recommended for reporting animal research, and Sex and Gender in Research

Laboratory animals

C57BL/6J male mice were purchased from Beijing Vital River Laboratory Animal Technology Co., Ltd. and kept under a 12/12h light/dark cycle, 24–26 °C, and 60% humidity.

Wild animals

No wild animals were used in this study.

Reporting on sex

Male C57BL/6J mice were used in this study.

Field-collected samples

The study did not involve field-collected samples.

| | |
|---|---|
| Ethics oversight | All experiment protocols were evaluated and approved by Institutional Animal Care and Use Committee (SIAT-IACUC-200320-YGS-TW-A1184) of Shenzhen Institutes of Advanced Technology, Chinese Academy of Sciences. |

Note that full information on the approval of the study protocol must also be provided in the manuscript.

## Flow Cytometry

### Plots

Confirm that:

- [x] The axis labels state the marker and fluorochrome used (e.g. CD4-FITC).
- [x] The axis scales are clearly visible. Include numbers along axes only for bottom left plot of group (a 'group' is an analysis of identical markers).
- [x] All plots are contour plots with outliers or pseudocolor plots.
- [x] A numerical value for number of cells or percentage (with statistics) is provided.

### Methodology

| | |
|---|---|
| Sample preparation | For flow cytometric analysis, fresh bone specimens were collected, and soft tissues attached onto the bone were removed. To obtain single cell suspensions, each specimen was crushed in the ice-cold HBSS buffer containing 2% FBS with mortar and pestle, followed by enzymatic digestion with collagenase (3 mg/mL), neutral protease (4 mg/mL), and DNAse (200 U/mL) at 37°C for 15 min. The digestion was quenched by addition of the HBSS buffer (Mg/Ca-free) containing 2% FBS and 40 µL of EDTA. After filtering using a 40 µm strainer, cells were collected via centrifugation and resuspended in PBS. Then, the cells were counted and adjusted to the density of 1,000,000 cells/100 µL in PBS. To determine cell viability, all the cells were labeled with Near-IR fluorescent reactive dye (Thermo, L34976) at 4°C for 30 min. After washing, the cells were blocked with anti-mouse CD16/32 antibody (Biolegend, 101320) at 4°C for 10 min. Then, an equal number of cells (density of 1,000,000 cells/100 µL) were subjected to immunostaining. |
| Instrument | CytoFLEX-S cytometer (Beckman Coulter) |
| Software | CytExpert software (Beckman Coulter) |
| Cell population abundance | *Describe the abundance of the relevant cell populations within post-sort fractions, providing details on the purity of the samples and how it was determined.* |
| Gating strategy | Viable single cell population were used for analysis. Staining boundaries were determined by comparing the fluorescence signals of unstained, single stained and isotype controls. |

- [x] Tick this box to confirm that a figure exemplifying the gating strategy is provided in the Supplementary Information.
